# Separation of small extracellular vesicles (sEV) from human blood by Superose 6 size exclusion chromatography

**DOI:** 10.1002/jev2.70008

**Published:** 2024-10-23

**Authors:** Jerome Nouvel, Gonzalo Bustos‐Quevedo, Tony Prinz, Ramsha Masood, George Daaboul, Tanja Gainey‐Schleicher, Uwe Wittel, Sophia Chikhladze, Bence Melykuti, Martin Helmstaedter, Karl Winkler, Irina Nazarenko, Gerhard Pütz

**Affiliations:** ^1^ Institute for Infection Prevention and Hospital Epidemiology Freiburg Germany; ^2^ Medical Center, Faculty of Medicine, University of Freiburg University of Freiburg Freiburg Germany; ^3^ Institute of Clinical Chemistry and Laboratory Medicine Freiburg Germany; ^4^ NanoView Biosciences Boston Massachusetts USA; ^5^ Department of General and Visceral Surgery Freiburg Germany; ^6^ IMITATE EM Core Facility Freiburg Germany; ^7^ Hahn‐Schikard Freiburg Germany; ^8^ German Cancer Consortium (DKTK) Partner Site Freiburg and German Cancer Research Center (DKFZ) Heidelberg Germany

**Keywords:** biomarker, extracellular vesicles, fast protein liquid chromatography, liquid biopsy, pancreatic cancer, single particle interferometric imaging, size exclusion chromatography

## Abstract

Extracellular vesicles (EVs) are valuable targets for liquid biopsy. However, attempts to introduce EV‐based biomarkers into clinical practice have not been successful to the extent expected. One of the reasons for this failure is the lack of reliable methods for EV baseline purification from complex biofluids, such as cell‐free plasma or serum. Because available one‐step approaches for EV isolation are insufficient to purify EVs, the majority of studies on clinical samples were performed either on a mixture of EVs and lipoproteins, whilst the real number of EVs and their individual specific biomarker content remained elusive, or on a low number of samples of sufficient volume to allow elaborate 2‐step EV separation by size and density, resulting in a high purity but utmost low recovery. Here we introduce Fast Protein Liquid Chromatography (FPLC) using Superose 6 as a matrix to obtain small EVs from biofluids that are almost free of soluble proteins and lipoproteins. Along with the estimation of a realistic number of small EVs in human samples, we show temporal resolution of the effect of the duration of postprandial phase on the proportion of lipoproteins in purified EVs, suggesting acceptable time frames additionally to the recommendation to use fasting samples for human studies. Furthermore, we assessed a potential value of pure EVs for liquid biopsy, exemplarily examining EV‐ and tumour‐biomarkers in pure FPLC‐derived fractions isolated from the serum of patients with pancreatic cancer. Consistent among different techniques, showed the presence of diseases‐associated biomarkers in pure EVs, supporting the feasibility of using single‐vesicle analysis for liquid biopsy.

## 1 INTRODUCTION

Extracellular vesicles (EVs) are natural subcellular structures, which are released by cells into the extracellular environment for regulatory purposes, such as modulation of microenvironment and intercellular communication. They consist of a lipid bilayer membrane and a hydrophilic core rich in functional biomolecules such as proteins and nucleic acids, whose composition depends on the EV origin (Yáñez‐Mó et al., 2015). Also, EV size can vary depending on their biogenesis. We focus in our manuscript on small extracellular vesicles, smaller 200 nm, separated from the larger particles by using a standard 0.22 µm PES filter (Jia et al., 2022). EVs play an integral role in a number of physiological and pathological processes. Blood plasma contains many bioactive components, including high concentrations of lipoproteins, proteins and metabolites. Purification of blood components is a tenuous task, especially when low‐abundant components like EVs are the target. Among particles, lipoproteins are the most abundant components (Daniels et al., 2009). In contrast to EVs, all lipoproteins consist of a hydrophobic lipid core covered by a single layer of phospholipids and one or more characteristic apolipoproteins. They can be classified according to their density into very‐low‐density lipoproteins (VLDL, <1.006 kg/L), intermediate‐density lipoproteins (IDL, 1.006–1.019 kg/L), low‐density lipoproteins (LDL, 1019–1.063 kg/L) and high‐density lipoproteins (HDL, 1.063–1.21 kg/L) (Winkler et al., 2003). VLDL, IDL and LDL contain a single copy of their core structure protein Apolipoprotein B100 (ApoB100), which is not interchangeable (Sigurdsson et al., 1975). In the fasting state, VLDL, secreted by the liver, are the largest lipoproteins with a median diameter of about 60 nm, whilst in postprandial state, very large (up to 1 µm) triglyceride‐rich chylomicrons (CM), carrying ApoB48, a truncated form of ApoB100, are secreted by the gut cells, and rapidly converted to CM remnants (∼70 to 150 nm) in the circulation (Hultin & Olivecrona, 1998). In contrast to ApoB100, apolipoprotein A1 (ApoA1), the characteristic apolipoprotein of HDL, can exchange between HDL particles and possibly other lipid structures, furthermore, one HDL particle may carry several ApoA1 molecules (Cavigiolio et al., 2010).

The concentration of lipoproteins in plasma is several orders of magnitude higher than the estimated number of EVs (Table ST1) (Clayton et al., 2019; Hartjes et al., 2019; Johnsen et al., 2019; Simonsen, 2017; Théry et al., 2018). Given overlapping size (LDL, VLDL and CM remnants) and density ranges (HDL), co‐isolation of lipoproteins and EVs from serum or plasma samples remains an unsolved technical challenge. One of the solutions, a sequential application of at least 2‐step‐separation according to the size and density, such as a combination of the density gradient purification and size exclusion chromatography (SEC), needs large sample volumes, is time‐ and efforts consuming, results in a low EV recovery, and is therefore incompatible with the large sample numbers needed in clinical studies. Among available single‐step isolation methods, such as ultracentrifugation (UC), precipitation and SEC, the latter gained attention as an easy‐to‐use, reliable way to remove protein contaminants, and partly lipoproteins (Holcar et al., 2021; Monguió‐Tortajada et al., 2019). Few studies performed so far demonstrated that application of different matrixes for SEC impact the quality of separation and recovery (Baranyai et al., 2015; Monguió‐Tortajada et al., 2019). Inspired by an example of successful separation of liposomes, which are similar in size to EVs, from animal lipoproteins by Fast Protein Liquid Chromatography (FPLC) using Superose 6 (S6‐FPLC) (Wiesner et al., 2009), we describe the use of S6‐FPLC for one‐step separation of EVs from majority of lipoproteins and bulk proteins from serum of healthy human donors and cancer patients in this manuscript.

## 2 MATERIAL AND METHODS

An overview of methods used for EV enrichment is provided in Figure S1.

### 2.1 Cell culture

Human breast cancer cell lines MDA‐MB‐361, MDA‐MB‐231 and fibrosarcoma cell line HT1080 and HT1080‐CD9‐GFP derivative were purchased from the ATCC cell culture collection (ATCC, Manassas, USA). Cells were cultured at 37°C, 5% CO_2_ and 95% humidity in antibiotics‐free Dulbecco's Modified Eagle's Medium (DMEM, Gibco/Thermo Fisher Scientific, Freiburg, Germany), supplemented with 10% foetal bovine serum (FBS, Thermo Fisher Scientific, Freiburg, Germany) and 20 mM L‐glutamine (Sigma‐Aldrich, Schnelldorf, Germany). For EV isolation, cells were seeded in 14.5 cm plates (5 × 10^7^ cells/plate) or T‐175 cm^2^ flasks (1 × 10^8^ cells/flask) and cultured until reaching 70%–80% confluence. Then the cells were washed twice with phosphate‐buffered saline (1 × PBS) to remove the remaining FBS, and cultured for 24 h (HT1080, HT1080‐CD9‐GFP), 48 h (MDA‐MB‐231) and 72 h (MDA‐MB‐361) in serum‐free cell culture medium. All plates were visually controlled by phase‐contrast microscopy for pyknosis and bacteria contamination. Cell viability ≥96% was mandatory for EV isolation as measured by an automated cell counter (Luna, Logos Biosystems, Villeneuve d'Ascq, France).

### 2.2 Enrichment of crude EV fractions, from cell culture supernatants using ultracentrifugation

Before EV purification from the cell culture supernatants by FPLC, small EVs were enriched by consecutive ultracentrifugation (cUC). For that, supernatants were collected from 20 × 14.5 cm plates in pre‐cooled 250 mL high‐speed centrifugation bottles (Neolab, Heidelberg, Germany), and centrifuged at 2000 × *g* for 30 min at 4°C, followed by 5000 × *g* for 45 min at 4°C, followed by 12,000 × *g* for 30 min at 4°C. Then the cell supernatants were filtered through 0.22 µm filtration membrane (Millex‐HP, Merck Millipore, Darmstadt, Germany) and concentrated to approximately 50 mL using a stirred‐cell (Bio‐Rad, Munich, Germany) supplemented with an ultrafiltration (UF) disc of 300 kDa cut‐off (Biomax®, Amicon, Millipore, USA). Finally, the EV‐containing fractions were sedimented using UC at 120,000 × *g* for 2 h at 4°C with an SW41 Ti swinging bucket rotor (Beckman Coulter, Fullerton, CA, USA). The pellets were re‐suspended in 200 µL ice‐cold 1× PBS and either directly used for FPLC, or stored at −80°C until further use.

### 2.3 Enrichment of BODIPY fluorescent EVs from cell culture supernatants using ultrafiltration

For a better EV detection and visualisation, fluorescent labelling was used as described earlier (Nazarenko et al., 2010). For that the cells were plated into T‐175 cm^2^ flasks and maintained as described above. Together with serum‐free medium, 0.5 mmol/L liposomal BODIPY‐cholesterol (BDPC, Biomol GmbH, Hamburg) were added. After incubation period of 2 h cells were washed and the cells were maintained for 2 more days in FCS free medium. For EV enrichment, cell culture supernatants were collected in pre‐cooled 50 mL Falcon tubes (BD Biosciences, CA, USA), centrifuged at 2000 × *g* for 15 min at 4°C and at 4000 × *g* for 45 min at 4°C to remove cell debris. Then the supernatants were transferred into Amicon™ Ultra‐15 centrifugation‐ tubes with the 100 kDa cut‐off (Merk, Darmstadt, Germany) and centrifuged at 4000 × *g* for 45 min to a final volume of approximately 1 mL. Concentrated supernatants were further used for the FPLC and density gradient centrifugation. By all steps, the samples were protected from the direct exposure to light to avoid bleaching of the fluorescent dye.

### 2.4 Enrichment of extracellular vesicles derived from HT1080‐CD9‐GFP cell culture supernatants

To corroborate the specific fractions containing EVs after the FPLC run, fluorescent EVs‐CD9‐GFP were used to spike serum samples from healthy donors and cancer patients. EVs‐CD9‐GFP were isolated from HT1080 cells stably transfected with a pcDNA3 plasmid harbouring CD9‐GFP, with CD9 fused to GFP at its C‐terminus. The cells were routinely monitored for CD9 and GFP expression using flow cytometry and Western Blotting. The supernatant was collected after 24 h and centrifuged at 800 × *g* for 5 min to remove cells and debris, followed by centrifugation at 5000 × *g* for 15 min to eliminate remaining cell debris and apoptotic bodies. Subsequently, the supernatant was filtered through a 0.22 µm PES syringe filter (Millex‐GP filter, MERCK) and concentrated using tangential flow filtration (TFF‐easy, Hansa Biomed) to reduce the volume to approximately 1 mL. Once the desired volume was achieved, size exclusion chromatography was performed (qEV 35 nm Generation 2, IZON) to enrich EVs in two fractions. Ten fractions of 400 µL were collected using an automatic fraction collector (AFC, IZON) with a void volume setting of 2.9 mL. Characterisation of fluorescent CD9‐GFP EVs is shown in supplemental Figure S2. Fractions 1 and 2 determined to be enriched in EV containing maximum particle number and CD9‐GFP protein were collected and concentrated using X‐Spinner (100 kDa cut‐off) columns. Final EV preparations were measured using fluorescent and scatter NTA to estimate the number of particles.

### 2.5 Blood samples collection, processing prior to EV isolation and storage

Blood samples were obtained from healthy volunteers and five patients diagnosed with pancreatic head carcinoma who were undergoing surgery for pancreatic cancer, following the provision of written informed consent. The study was approved by the local Ethics Committee of the University of Freiburg (EK 371/14; EK 563/17). For venous blood samples, 9 mL serum Monovettes (Sarstedt AG & Co. KG, Nuembrecht, Germany) were used. After clotting for 30 min at RT, samples were centrifuged twice for 10 min by 2500 × *g* at RT, each time transferring supernatant into a fresh tube. The remaining platelets and haemolysis were controlled according to the clinical diagnostics routine at the Medical Centre University of Freiburg. *T* = 1 Tsd/µL indicates increased levels of remaining platelets and was characteristic for the whole cohort of patients with pancreatic cancer counting over 100 patients. Therefore, an additional centrifugation step at 2500 × *g* and filtration using 0.22 µm filter were included prior to EV isolation from these samples.

Unless stated otherwise, samples from healthy donors were taken after overnight fasting. For postprandial samples, a standard fat meal of 1 g TG/kg bodyweight in a cream drink was digested after overnight fasting (Werner et al., 2014).

### 2.6 Isolation of lipoproteins from the blood samples

For isolation of lipoproteins, fasting plasma samples from healthy donors were subjected to preparative sequential density ultracentrifugation with a target density less than 1.006 kg/L for VLDL, between, 1.006 and 1.019 kg/L for IDL, between 1.019 and 1.063 kg/L for LDL, between 1.063 and 1.21 kg/L for HDL as previously described (Havel et al., 1955; Werner et al., 2014). These isolated lipoproteins were used to calibrate the S6‐FPLC retention times (Figure S3).

### 2.7 Preparation of liposomes

Liposomes for S6‐FPLC calibration were prepared by extrusion (MacDonald et al., 1991) using (1,2‐di‐(9Z‐octadecenoyl)‐sn‐glycero‐3‐phosphocholine, cholesterol and (2‐distearoyl‐sn‐glycero‐3 phosphoethanolamine‐N‐[methoxy (polyethylene glycol)‐2000) in the molar ratio 50/40/10. Briefly, all lipids were dissolved in chloroform and mixed, the chloroform was then removed and lipids were dried in vacuum. The dry lipid film was hydrated with a HEPES buffer (118 mM NaCl, 4.74 mM KCl, 1.2 mM MgCl_2_, 0.59 mM KH_2_PO4, 0.59 mM Na_2_HPO4, 10 mM HEPES, pH 7.4) at 50°C by rotation. To obtain liposomes with a size of approximately 100 nm, the lipid suspension was extruded using extruder LiposoFast Liposome Factory (Merck, Darmstadt, Germany) nine times through a polycarbonate membrane with 200 nm pore size, and afterwards 21 times through a membrane with 100 nm pore size (Avestin Europe GmbH, Mannheim, Germany). Bodipy‐cholesterol‐enriched liposomes were produced as described above consisting of 1‐palmitoyl‐2‐oleoyl‐glycero‐3‐phosphocholine/cholesterol in molar ratio 70/30 using TopFluor Cholesterol. Liposome concentration was determined with ammonium ferrothiocyanate (Stewart, 1980), liposome size was determined by the dynamic light scattering as described before (Ngoune et al., 2016). All lipids were obtained from Avanti (Avanti Polar Lipids, Alabaster, USA).

### 2.8 EV isolation using Superose® 6 fast protein liquid chromatography

FPLC was performed on a BioLogic DuoFlow (Bio‐Rad, München, Germany) FPLC system equipped with a Tricorn® column (10 mm diameter, 600 mm length) loaded with Superose® 6 prep grade (GE Healthcare, Freiburg, Germany). The column was packed according to the manufacturer's instructions by the aid of a packing reservoir. Quality control was done with acetone.

A sample volume of 250 µL was applied each time. The running buffer (RB) consisted of 1 × PBS, supplemented with sodium azide (200 mg/L) and EDTA (372.3 mg/L). Serum samples were diluted 1:4 (v:v) with PBS to achieve a total protein load <25 mg as recommended by the manufacturer. For recovery, after each run the column was rinsed with three column volumes of PBS and cleaned with 0.1 N NaOH every five runs. Sample separation was performed at RT (∼20°C) with a flow rate of 0.2 mL/min. Fractions (1 mL) were collected after 5 mL void volume. The optical density (OD) of the eluate was measured at 280 nm. Chromatograms were analysed using the BioLogic DuoFlow‐Demo Chromatography 5.0 Software (Bio‐Rad). After each run, fractions were used directly for the downstream analysis, or stored at −80°C.

### 2.9 Density of purified small EVs (sEV) isolated by FPLC using sodium bromide density gradient

#### 2.9.1 Density of purified small EVs (sEV) isolated by FPLC using sodium bromide density gradient

The EV‐enriched fractions F14+15, F16+17 and F18+19 were combined and their distribution in a sodium bromide density gradient (1.0064–1.47 g/mL) was investigated. To prepare the density gradient, 11.45 g NaCl; 0.5 g NaN3 and 0.1 g EDTA were dissolved in 1 kg aqua dest. To 200 mL of this solution either 39.12 g of NaBr (*d* = 1.1468 g/mL, NaBr solution 1) or 153 g NaBr (*d* = 1.47 g/mL, NaBr solution 2) were added. In a 6 mL Beckmann ultracentrifugation tube, 2 mL of NaBr solution 2; 2 mL of NaBr solution 1 and 2 mL of respective FPLC fractions were carefully layered. Samples were centrifuged in a SW 50.4 Ti swinging buckets rotor (Beckman Coulter, Fullerton, CA, USA) for 4 h by 40,000 × *g* at 4°C. The resulting density gradient was fractionated, in each fraction, the BDPC fluorescence (exc./em. 495/507 nm) was determined and the density was measured by refractometry (Carl Zeiss, Germany).

### 2.10 Recovery of EVs and spiking experiment

To measure recovery of the S6‐FPLC, fluorescent EVs‐CD9‐GFP enriched by SEC as described above were used. Concentration 10^10^ particles were adjusted to the final volume of 300 µL if not otherwise indicated, from which 250 µL were injected for the isolation. Fractions F13 to F40 were measured by NTA in scatter and fluorescent mode, and particle numbers in F14‐F17, showing to contain CD9‐GFP signal were summarised to calculate the recovery of purified EVs. For the spiking experiment, serum samples of cancer patients or healthy donors (200 µL) were mixed with 100 µL 1 × 10^10^ CD9‐GFP EVs and run by S6‐FPLC.

### 2.11 Determination of EV‐enriched and lipoprotein‐enriched fractions using fluorescence‐linked FPLC

For identification of EV‐enriched and lipoprotein‐enriched fractions, S6‐FPLC samples were incubated with anti‐CD63‐APC (exc./em. 633/660 nm) (Biolegend, 353007) and anti‐ApoB‐FITC (exc./em. 493/528 nm) (Abcam, ab27637) or anti‐CD9‐PE (Biolegend, 312105) 1:50 (v/v) for 1 h at 4°C before loading. After chromatography as described above, triplicates of each fraction were analysed for either 633 nm fluorescence for allophycocyanin (APC), 532 nm fluorescence for Phycoerythrin (PE) or 493 nm fluorescence for fluorescein (FITC) using a microplate reader Varioskan Flash LemiSens (Thermo Fisher Scientific, MA, USA) equipped with Skan It 2.4.5 Software. Additionally, samples which were incubated with PE‐conjugated anti‐human CD9 (312105, Biolegend) only, were examined using NTA in scatter and fluorescent mode.

### 2.12 Characterisation of particles by nanoparticle tracking analysis (NTA) and data analysis

The concentration and size distribution of the EVs/particles from the different fractions isolates from SEC/FPLC were measured immediately after isolation and/or enrichment using the Nanoparticle Tracking Analysis (NTA) instrument Zetaview QUATT (Particle Metrix, Meerbusch, Germany). For that, the instrument was calibrated as shown in Figure S4. For measurement, the samples were diluted in 0.1× PBS; measurements were performed in scatter and fluorescent‐488 nm modes. All fractions were measured first in scatter mode, using the 488 nm laser (Figure S4) to visualize de particles. Then for the measurement of particles in fluorescent mode, the 500 nm filter was used for the visualisation of the emission signal.

Isolation of particles by FPLC obtaining the separation of the sample in 40 fractions. Fractions were measured by nanoparticle tracking analysis (NTA) to calculate the median size of each fraction and the estimation of the particle concentration. Each fractions plot represents the size distribution having in the y‐axe the particle concentration in [particles/mL] and in the x‐axe the particle size in [nm]. The selected plots correspond to the fractions where the main particles are contained. Fraction 13 is used as the control to show that no particles or low concentrations are visible in earlier fractions. The isolation consisted of the separation of EVs from VLDL, LDL, HDL and soluble proteins, starting from the EVs in fraction 16, and fractions 18, 25, 32 and 34 respectively.

### 2.13 Western blot

FPLC fractions (700 µL) were concentrated to a final volume of 200 µL using Merck™ Amicon™ Ultra‐0.5 Centrifugal Filter Units with 10 kD membrane. Western blotting was performed using the western blotting system Criterion™ Blotter (Bio‐Rad) according to the manufacturer's instructions. 30 µL of concentrated FPLC fraction sample was loaded on either a 10% (for EV marker) or on 8% (for ApoB) SDS PAGE gel and then transferred to polyvinylidene fluoride (PVDF) membranes. The membranes were blocked in 5% skim milk for 1 h and incubated overnight with primary antibody solution (anti‐CD63 1:100, anti‐Alix 1:100, anti‐ApoB 1: 2000) at 4°C. For signal visualisation secondary antibodies conjugated with horse radish peroxidase (HRP) were used. The blots were developed using a luminol‐based enhanced chemiluminescent (ECL) substrate for detection of HRP activity and analysed with Bio‐Rad Chemi doc XRS system using the Quantity One 4.6.6 software. All antibodies used and corresponding dilutions are annotated in Table S3.

### 2.14 Beads‐assisted flow cytometry

To determine the portion of EVs, harbouring either universal EV proteins, such as CD63, or cell‐specific biomarkers, such as EpCAM for epithelial cells or Tspan8 for pancreatic cancer cells, beads‐assisted flow cytometry was used. For that isolated EVs were incubated with latex beads in 1 x PBS; free aldehyde and sulphate groups were blocked with 1 M glycine. Latex beads were pelleted and incubated subsequently with a primary antibody conjugated with a fluorescent epitope for 1 h. Then the beads were washed twice with 1 × PBS, and measured using, the threshold of 80,000 FSC‐H (T1) and 10 SSC‐H (T2); 50,000 events were recorded for the sample.

### 2.15 Transmission electron microscopy (TEM)

TEM was performed for confirmation of EV‐enriched fractions. 10 µL of each fraction was loaded on a 300‐mesh copper grid and fixed with 1% glutaraldehyde. After washing with double distilled water the samples were negatively stained with 10 µL drop of 1% uranyl acetate. The images were taken using an electron microscope (LEO 906 E, Zeiss, Oberkochen, Germany) using SIS software (Olympus, Hamburg, Germany).

## 3 RESULTS

### 3.1 Calibration of S6‐FPLC and validation of EV‐separation from cell culture supernatant

**FIGURE 1:**
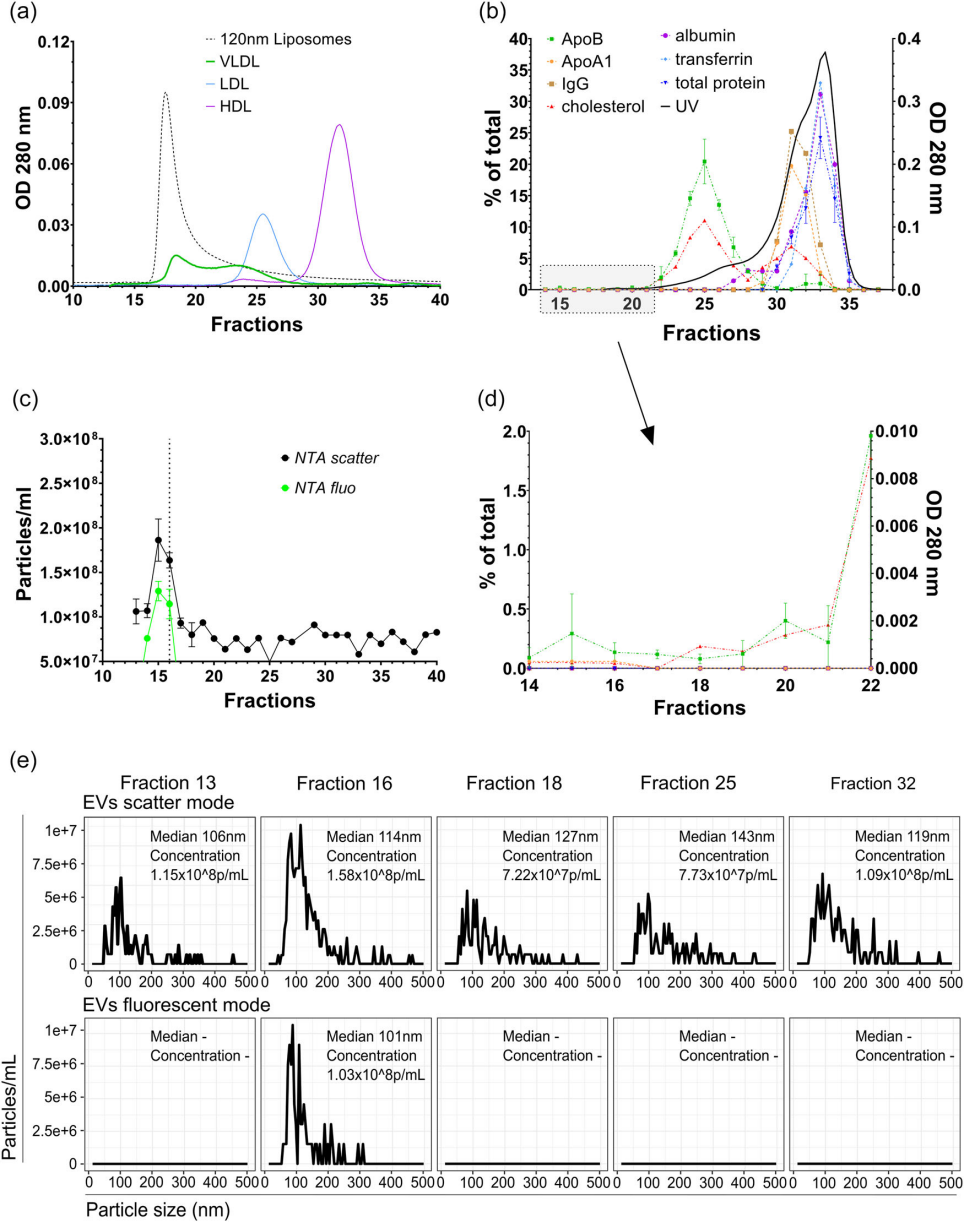
Calibration of S6‐FPLC. (a) S6‐FPLC chromatograms (OD 280 nm measurement) with either liposomes (*d* = 121 nm) or isolated lipoprotein species VLDL, LDL and HDL (*n* = 3). (b) Chromatogram of healthy human serum (Black solid line: OD measurement) and distribution of abundant human serum proteins in S6‐FPLC (coloured/dotted lines) (*n* = 3). (c) Fluorescent EVs derived from CD9‐GFP transfected HT1080 cells (10^10^ particles) were subjected to S6 FPLC and fractions were analysed by NTA in scatter and fluorescent mode (*n* = 2). (d) Zoom in from B in the range where EVs are. (e) Details of NTA measurement from C. (representative image).

As the first step, the S6‐FPLC setup was calibrated using liposomes and lipoprotein species isolated by sequential density UC (Figure 1a). The liposomes with an average hydrodynamic diameter of 121 ± 20 nm peaked in fraction 17, VLDL with a diameter of 30–80 nm in fraction 18–25, LDL (18–25 nm diameter) in fraction 25–28 and HDL (5–15 nm diameter) in fraction 30–35, showing a baseline separation between liposomes and LDL/HDL, but not necessary between liposomes and VLDL. Similarly, when protein distribution was measured using human serum from a healthy donor (Figure 1b), ApoB, corresponding to VLDL and LDL, peaked in fraction 25, ApoA1, corresponding to HDL, in fraction 31, whilst cholesterol peaked in fraction 25 and 35. Only minute amounts of ApoB were found in fractions 15–18, significantly increasing from fraction 20 onwards (Figure 1d). High abundant plasma proteins peaked in fractions 31 (IgG) or fraction 33 (albumin, transferrin, total protein), with no signal above LOD in fractions 15–18. Recovery for plasma proteins was between 46.8% (ApoA1) and 87.7% (albumin) (Figure S3). EVs containing CD9‐GFP, derived from cell culture supernatant by cUC in combination with commercial SEC, were subjected to S6‐FPLC (Figure 1c). EVs peaked from F14 to F17 with a maximum peak in F16 as stated by NTA in scatter and fluorescent mode (Figure 1e). In fluorescence mode, no signal was recorded by NTA beyond respective fractions F14‐F17, in contrast to scatter mode, where a residual background was measured in all FPLC fractions. The median size of EVs was 114 nm (scatter) or 101 nm (fluorescence) as expected. Recovery for isolated EVs (1 × 10^10^) as measured by NTA was 7.3 (± 0.2) % in scatter mode and 3.6 (± 0.7) % in fluorescence mode. Thus particles in the size range of sEVs should be separated from plasma lipoproteins by S6‐FPLC.

**FIGURE 2:**
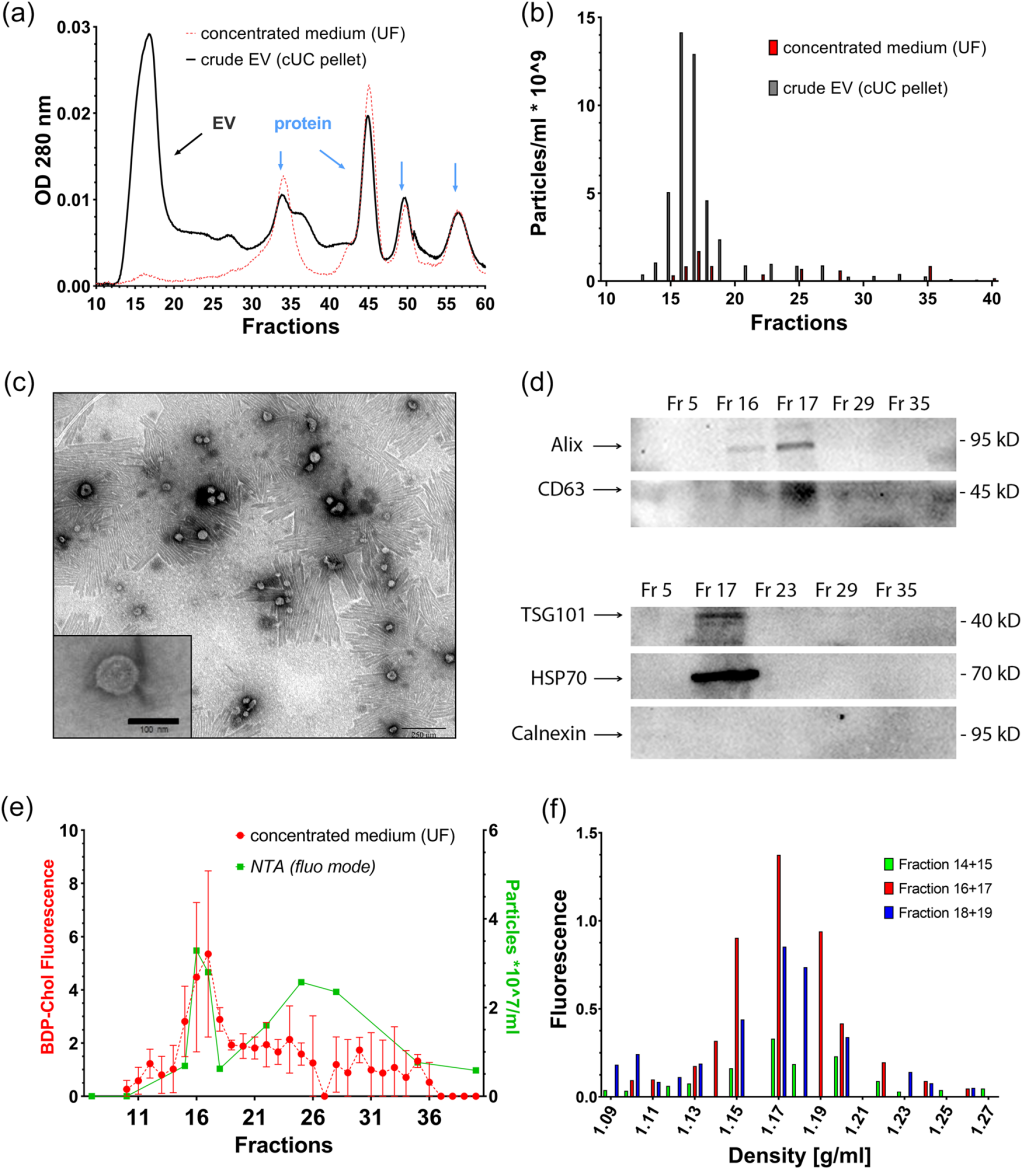
Fractions F16‐F18 were determined as EV‐containing fractions after separation of cell culture supernatants by S6‐FPLC. To determine the EV‐containing fractions, 250 µL of crude EV fractions enriched by cUC or UF were loaded on a S6‐SEC column of 9 × 600 mm with a flow rate of 0.2 mL/min phosphate buffered saline (*n* = 4). (a) Typical chromatogram of cell culture supernatants (black line: cUC enriched supernatant of MDA‐MB‐361 cells; red dotted line: UF enriched medium of MDA‐MB 231 cells. (b) Analysis of particle number in FPLC fractions using NTA (scatter mode), typical measurement. (c) Representative TEM image of F17, containing majority of CD‐63 positive particles, most probably EVs; wide field (scale bar 250 nm) and zoomed‐in picture (scale bar 100 nm). (d) Immunoblotting of EV‐specific markers Alix, CD63, HSP70, TSG101 as EV‐ and Calnexin as ER‐marker (*n* = 3‐4). (e) Measurement of BODIPY‐fluorescence in S6‐FPLC fractions by fluorescence spectrometry (red line) and NTA in fluorescence mode (green line) of UF cell culture supernatants after labelling of 2 × T 175 cm^2^ MDA‐MB 231 cell culture plates with BODIPY‐Cholesterol (*n* = 3). (f) Particle density analysis of S6‐FPLC derived BODIPY‐Cholesterol labelled particles in fractions F14‐19 by NaBr density gradient UC and subsequent fluorescence spectrometry of respective density layers (*n* = 3).

Next, cell culture supernatant concentrated by UF or crude EV fractions enriched by cUC were subjected to S6‐FPLC (Figure 2a). Besides several protein‐containing peaks, an additional 280 nm UV signal was detected in the fractions F15‐F18, where small EVs might be expected (Figure 2a). Supporting that, the corresponding signal in F15‐F18 for the cUC enriched sample (Figure 2a, black line) was several fold higher than the signal for UF concentrated medium (Figure 2a, red line), whilst non‐EV protein signals remained similar. Next, the enrichment of EVs in the fractions F15‐F18 was verified in several steps. The NTA analysis revealed a high number of particles in the fractions F13‐F19, whilst other fractions contained only minute numbers of particles (Figure 2b). As expected, the number of particles in cUC‐enriched samples in these fractions was several fold higher than in the conditioned medium after UF. Obtained S6‐FPLC fractions were further characterised by TEM, revealing EV‐like high‐density structures around 100 nm in diameter surrounded by a membrane bilayer (Figure 2c) in the fraction 17, but not in the fractions F11, F29, F35 tested as a control (Figure S5). This finding was supported by Western Blotting revealing the strongest signal for EV‐ characteristic proteins CD63, Alix, TSG101 and HSP70 in the fraction F17, but not for Calnexin tested as a negative control (Figure 2d; Figure S6).

To obtain cell derived membrane labelled vesicles, cell membranes were labelled with BDPC. After 2 days, cell culture supernatants containing respective fluorescent cell derived vesicles were concentrated by UF and subjected to S6‐FPLC. The strongest fluorescence signals were observed in F16‐F17 (Figure 2e, red line). NTA performed in fluorescence mode supported EV localisation in the fractions F16‐F17, showing maximum fluorescent labelled particles in these fractions (Figure 2e, green line). Fractions F14‐F19, containing fluorescent particles, were further examined by density gradient centrifugation (Figure 2f) (Wiesner et al., 2009). Maximum fluorescence intensity was observed in the fractions with the EV characteristic density range of 1.15–1.19 g/mL (Nazarenko et al., 2010; Onódi et al., 2018), whilst no fluorescence above background was seen in densities < 1.13 g/mL. Highest intensities were observed in F16‐F17, supporting that the majority of BDPC labelled EVs was located in these fractions. For F14‐F18, no fluorescence above background level was detected in the VLDL/LDL‐specific densities < 1.062 g/mL or beyond 1.21 g/mL, corresponding to lipid‐rich particles and protein‐bound BDPC, respectively (Figure 2f). Taken together, the particles in F16/F17 are classified as EVs by EV specific biomarkers, size and density.

### 3.2 Separation of EVs and lipoproteins from healthy and cancer patient serum by S6‐FPLC and the impact of nutritional status of a donor

**FIGURE 3:**
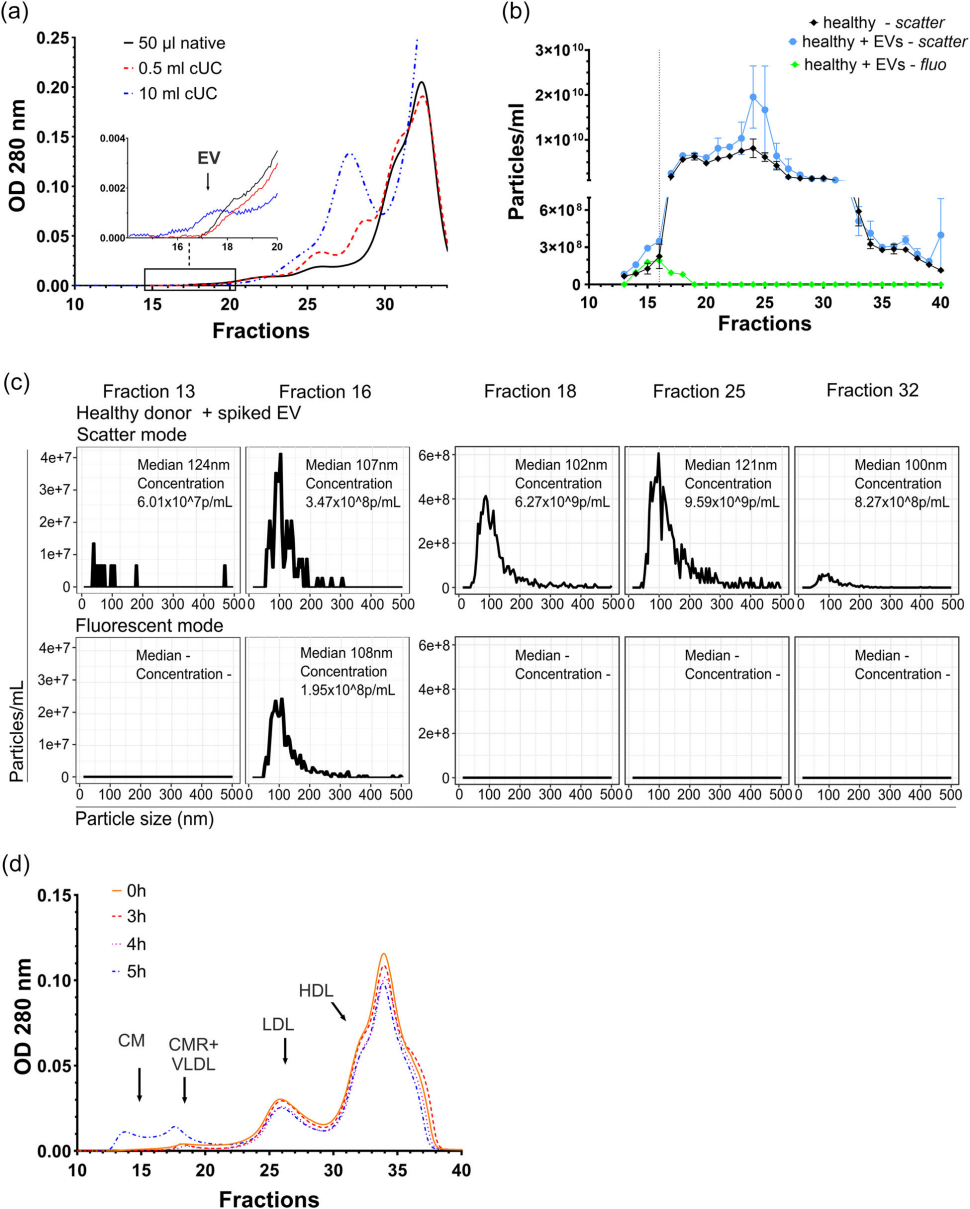
Separation of EVs and lipoproteins from healthy human serum using S6‐FPLC allows enrichment of EVs in fraction F16 and F17 under fasting conditions. (a) Overlay of representative S6‐FPLC chromatograms (OD 280 nm measurement) of undiluted fasting serum and cUC‐concentrated samples derived from 0.5 to 10 mL fasting serum of healthy donors. Enlargement emphasises the EV‐containing S6‐FPLC fractions 16–18. (b) NTA analysis (mean and s.d.) of S6‐FPLC fractions of fasting serum derived from healthy donors (black line). Some samples (blue and green line) were spiked with fluorescent EVs derived from CD9‐GFP transfected HT1080 cells (10^10^ particles). (c) Details of NTA measurement from B (representative image). (d) Overlay of representative S6‐FPLC chromatograms of postprandial serum of a healthy donor 0, 3, 4 and 5 h after ingesting a standard fat meal (1 g TGs/kg body weight). All experiments were done in biological triplicates.

One of the important quality parameters of an EV isolation method is its ability to separate pure EVs from complex biofluids, such as blood. To evaluate the performance of S6‐FPLC in EV separation, 250 µL diluted human serum (1:4 v/v) of a fasting healthy individual was subjected to S6‐FPLC (Figure 3a, black line). OD signals were recorded in the characteristic fractions for LDL, HDL, protein and to a very low extent in the size range of VLDL (Figure 3a). In contrast, no OD signal was detected in the EV‐characteristic fractions F16‐F17, reflecting the much lower number of EVs than lipoproteins in human serum. Concentrating samples of 0.5 or 10 mL human serum using cUC before the S6‐FPLC separation revealed a small peak in F16‐F17 that was detected in 10 mL samples, but not in 0.5 mL samples due to the limited sensitivity of 280 nm optical absorption measurement (Figure 3a).

To evaluate particle abundance of EV‐ versus non‐EV fractions in the serum of healthy individuals, NTA was performed (Figure 3b). To increase the accuracy of the measurements, the NTA instrument was calibrated using silicon dioxide nanoparticles, which have a refractive index closer to the refractive index of EVs, 1.45 and 1.42, respectively (van der Pol et al., 2014), preliminary validated using electron microscopy (Figure S4). With regard to the total particle number recorded in NTA, less than 1% of particles were in the fraction range of EVs, whilst the overwhelming number of particles detected were in the range of LDL and HDL (Figure 3b). When serum was spiked with fluorescent EVs derived from cell culture, fluorescent particles were detected between F14‐F18, peaking in F16 (Figure 3b, green line), whilst in scatter mode, particle numbers increased in F14‐F16 (Fig3B, blue line).

Again, in fluorescence mode, no signal was recorded by NTA beyond sEV containing fractions F14‐F17, in contrast to scatter mode, where significant numbers of particles were recorded in F18 onwards even though, smaller lipoproteins are less visible by the NTA method optimised to record particles in the size range of ∼100 nm. The median size of EVs was 107 nm (scatter) or 108 nm (fluorescence) as expected for sEVs (Figure 3c).

To evaluate the impact of non‐fasting conditions on EV sampling, healthy volunteers performed a standard lipid tolerance test by ingesting about 70 g of TGs with a single meal. Serum samples that were taken after certain time points were subjected to S6‐FPLC (Figure 3d). A significant postprandial signal was detected as early as F12 in serum samples taken 4–5 h after food intake (Figure 3, blue line) and lasted until F20, showing two maximums at F14 and F18. These postprandial lipoprotein peaks overlapped with the EV‐containing fractions F16‐F17, indicating contamination of EV samples with postprandial lipoproteins.

**FIGURE 4:**
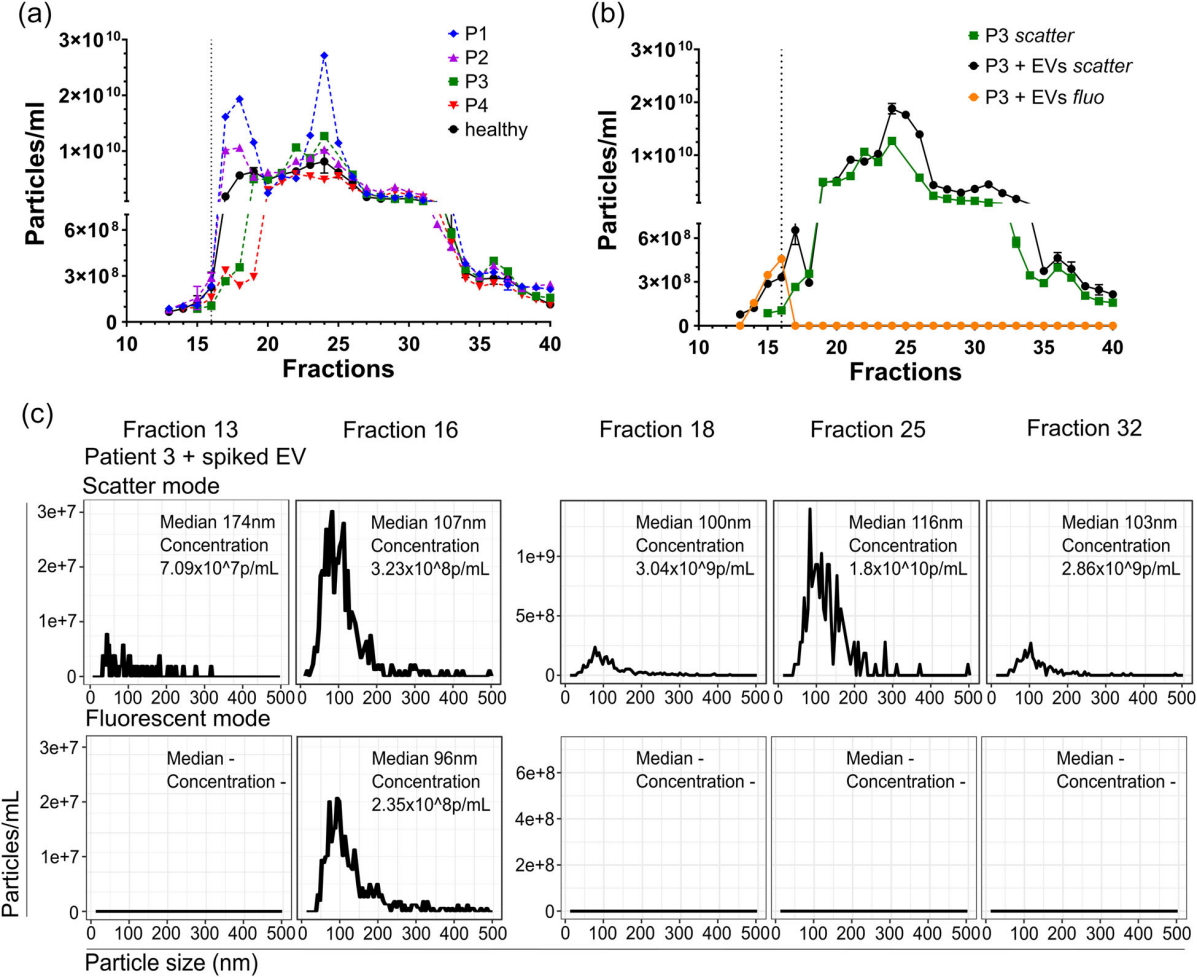
Separation of EVs from cancer patients serum using S6‐FPLC (a) NTA analysis (mean and s.d.) of S6‐FPLC fractions of fasting serum derived from different prostate cancer patients (coloured lines) or healthy donor (black line). (b) Patient sample 3 spiked with fluorescent EVs derived from CD9‐GFP transfected HT1080 cells (10^10^ particles) (*n* = 2). (c) Details of NTA measurement from B. (representative image).

When pancreatic cancer patient samples were used for S6‐FPLC separation of EVs, overall only a minor portion of particles was found in the EV containing fractions F15‐F17, as already observed for healthy subjects (Figure 4a). Significant differences in particle numbers were observed for the EV containing fractions F16/F17. Compared to healthy serum, significantly higher particle numbers were recorded for two cancer patient samples, whilst other samples showed lower EV counts. Again, the spiking of cancer patient serum with fluorescent EVs from cell culture showed fluorescent particles in F15‐F17 and an increase in particle numbers in respective fractions in scatter mode (Figure 4b). Again, in fluorescence mode, no signal was recorded by NTA beyond sEV containing fractions F14‐F17, in contrast to scatter mode, where significant numbers of particles were recorded in F18 onwards (Figure 4b,c).

### 3.3 Advantages of the fluorescence‐linked S6‐FPLC for separation of EV‐ and non‐EV blood components and biomarker detection

**FIGURE 5:**
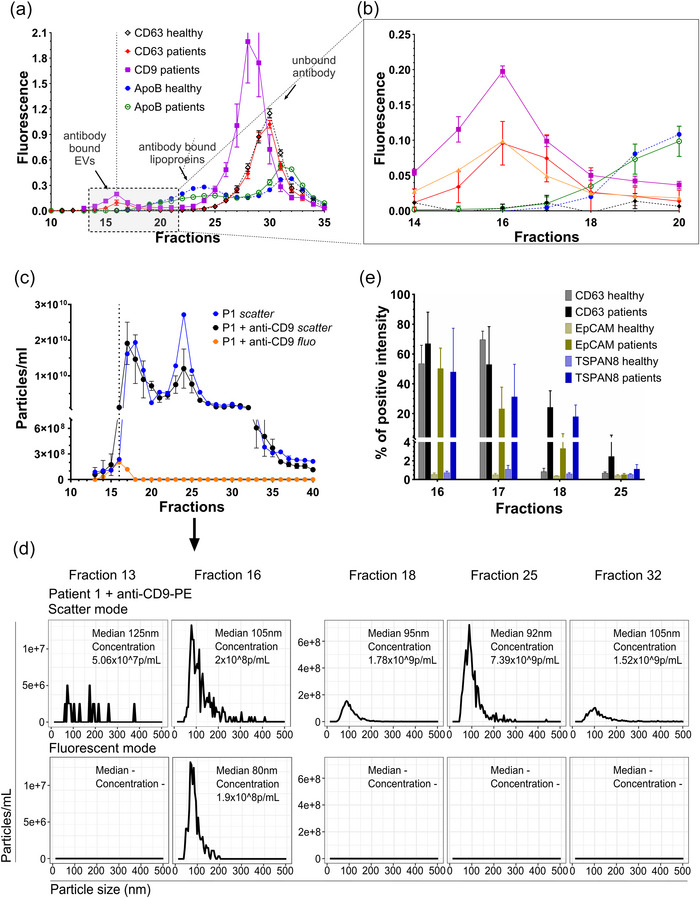
Separation of EVs and lipoproteins from human cancer patient serum using S6‐FPLC and detection of EV biomarker in fractions F16 and F17. (a) FL‐S6‐FPLC prior to S6‐FPLC, anti‐CD63‐APC (633/660 nm) and Anti‐ApoB‐FITC (493/528 nm) or anti‐human CD9‐PE (532/572 nm) were added to cUC enriched serum of cancer patients (pink/red/green lines, *n* = 3) or healthy donors (black/blue lines, *n* = 4). Obtained FL‐S6‐FPLC fractions were analysed by fluorescence spectrometry. (b) Zoom in from B in the range where EVs are. (c) Patient P1 serum spiked with fluorescent antibody against CD9 and measured by NTA (*n* = 2). (d) Details of NTA measurement from C (representative image). (e) Beads‐assisted flow cytometry using latex beads of the S6‐FPLC fractions derived from healthy donors or cancer patients using (*n* = 3 healthy, 4 patients).

To allow a more sensitive and reliable detection of EV‐containing fractions than OD measurements, fluorescence‐linked S6‐FPLC (FL‐S6‐FPLC) was developed. As IgG have significant longer elution times than EVs (Figure 1b), adding antibodies directed against EV biomarker like CD63 or CD9, S6‐FPLC should allow a one‐step separation and EV biomarker detection. First, the separation of unbound and EV‐bound antibodies was proven using EVs isolated from cell culture supernatant. Although the unbound antibody was detected in the fractions F26 onwards, antibody labelled EVs were localised in the fractions F16‐F17 as expected (Figure S7). The applicability of this approach was tested on human serum samples derived either from healthy donors or from pancreatic cancer patients. Prior to S6‐FPLC, samples were incubated with fluorescent antibodies, either against CD63, CD9 or against ApoB as control for potential contaminating CM and VLDL in F15‐F17. Using different fluorophores (anti CD63‐APC; anti‐CD9‐PE and anti‐ApoB‐FITC), respective signals can be analysed from a single run (Figure 5a). In both, healthy and tumour samples, the ApoB‐FITC signal was absent until F18, peaking in F24, indicating that the EV‐fractions F16‐F17 are almost lipoprotein‐free (Figure 5a, green or blue line). However, whilst the anti‐CD63‐APC or anti‐CD9‐PE fluorescence were well‐detectable in the fractions F16‐F17 in tumour samples (Figure 5b, red, violet lines), it remained at the detection limit in the samples of healthy donors. These measurements were further supported using fluorescent NTA. For that NTA‐analysis in scatter and fluorescent modes was performed with the 13 onwards S6‐FPLC fractions from the cancer patient serum incubated with the anti‐CD9‐PE antibody (Figure 5c). Consistent with the fluorescence measurement, fluorescent particles were observed only in F15‐F17, supporting the presence of CD9‐positive EVs in these fractions (Figure 5b, orange line; Figure 5c). Consistent with the data observed in the spike‐experiments showing in the Figure 4, scatter mode showed significantly higher numbers of particles in F18 onwards, whilst the fluorescent particles were detected only in the fraction F16 (Figure 5d).

Additionally, we tested presence of cancer‐related biomarkers in the fractions of interest. For that beads‐assisted flow cytometry was used as a batch method allowing to increase sensitivity of detection. As biomarkers, we selected EpCAM and Tspan8; CD63 was used as a positive control (Figure 5e). Analysis of S6‐FPLC fractions revealed co‐localisation of CD63, EpCAM and Tspan8‐specific fluorescence in the fractions F16, F17, F18, with a maximum in F16. No signal was detected in the LDL fraction F25. This observation confirms the concentration of putative EVs in the fractions F16 and F17, consistent with the data shown above. The absence of EV‐specific signal in the major LDL fraction F25 indicates that LDL, if properly separated from EVs, do not contain EV‐associated membrane proteins.

As an additional control, we tested the presence of soluble proteins Factor VII and CRP in the S6‐FPLC fractions, demonstrating their presence in the protein fractions but not in the EV fractions (Figure S8), supporting the quality of separation of EV‐ and non‐EV components by the S6‐FPLC. Taken together, S6‐FPLC allows an excellent separation of blood‐derived EVs from proteins and lipoproteins for biomarker analysis preferably, when fasting samples are used.

### 3.4 Recovery of EVs and estimation of EV count in human serum

A major drawback of the described S6‐FPLC methods is a significant dilution of samples. If 1 × 10^10^ cell culture‐derived EVs were injected, the recovery of isolated EVs as measured by NTA was 7.3 (± 0.2) % in scatter mode and 3.6 (± 0.7) % in fluorescence mode (Table 1, line 1). Similar recovery rates were obtained when human serum was spiked with a comparable number of CD9‐GFP‐positve EVs (7.3 ± 1.2 %) as shown in Figures 4 and 5. Recovery was similar when similar numbers of liposomes were injected (Table 1, line 4). However, the recovery increased if higher amounts of EVs were injected. Thus, application of 4.6 × 10^10^ particles results in 12.5% recovery; whilst increasing the number to 1 × 10^11^ allowed to increase the recovery upto 36% (Table 1, lines 3 and 5, respectively). Commercial size exclusion chromatography columns exhibited similar tendency showing recovery range of 30% if 1 × 10^10^ were used, which was increased upto 60% if 1 × 10^11^ EVs were passed through the column. Consistent with the observation, recovery of EVs was significantly lower than recovery of highly abundant serum components including transferring, albumin or lipoproteins (Figure S3).

**TABLE 1:** Recovery of particles after size exclusion chromatography.

Procedure	Loaded particles	Recovery (%)	Recovery s.d. (%)	NTA
S6‐FPLC/EVs	1.0*10^10^	7.3	0.2	Scatter
S6‐FPLC/EVs	1.0*10^10^	3.2	0.7	Fluo
S6‐FPLC/EVs	4.6*10^10^	12.5	n.d.	Scatter
S6‐FPLC/ liposomes	1.0*10^10^	6.7	n.d.	Scatter
S6‐FPLC/EVs	1.1*10^11^	36.0	5.2	Scatter
IZON‐SEC/EVs	1.0*10^10^	30.2	6.2	Scatter
IZON‐SEC/EVs	3.9*10^11^	61.2	n.d.	Scatter

EVs were harvested from HT1080‐CD9‐GFP cells and liposomes were prepared as described. Particle numbers were measured by NTA and the desired particle numbers were adjusted by dilution with PBS. Either S6‐FPLC or commercial SEC (qEV 35 nm Generation 2, IZON) were used. Respective fractions were collected (S6‐FPLC F15‐F17/IZON F1‐F2, Figure S6) and particle numbers were measured by NTA again to calculate recovery (*n* = 2).

Similar phenomenon was observed by ultracentrifugation. Thus, about 1.9 (± 0.4) × 10^9^ particles/mL were counted in F17 if healthy serum was processed directly by S6‐FPCL. However, if ultracentrifugation was performed before in order to concentrated a sample, about 2.8 (± 0.8) × 10^8^ particles/mL were detected in F17 if 0.5 mL of human serum was subjected to ultracentrifugation, whilst 10 mL human serum yielded about 3.8 (± 0.1) × 10^9^ particles/mL in F17 (Figure S9), indicating significant loss of particles due to ultracentrifugation (cUC samples).

Subtracting the measured fluorescent EV number from the total measured EV number in scatter mode (F15‐F17) and assuming, that recovery for serum EVs is similar as recovery for spiked fluorescent EVs, the total EV number in human serum was estimated to 7.6 (± 4.7) × 10^10^ particles/mL.

## 4 DISCUSSION

### 4.1 S6‐FPLC for EV separation

Robust and reproducible isolation of high quality EV samples is mandatory for their use as clinical biomarker. Although in some biofluids like urine, EVs represent the main vesicle compound (Erdbrügger et al., 2021), obtaining pure EV samples from such complex biofluids as serum or plasma is much more challenging. Concentrations of the major lipoproteins LDL and HDL are up to six orders of magnitude higher than EVs in human plasma, and even low abundant human lipoproteins such as VLDL have significantly higher concentrations than do the EVs. HDL are the smallest lipoproteins but have similar densities as EVs. LDL are somewhat smaller in diameter than EVs, but postprandial CM remnants and large VLDL exhibit overlapping size ranges (Table 1) (Simonsen, 2017). High abundancy and these similarities in physical properties lead to a common co‐purification of EVs and lipoproteins by using conventional simple one‐step isolation methods (Brennan et al., 2020; Onódi et al., 2018; Pang et al., 2020; Yuana et al., 2014).

SEC won recognition for the separation of EVs and non‐EV proteins as a reliable and easy‐to‐use method; however, the approaches described so far were not satisfactory in separation of EVs and lipoproteins (Baranyai et al., 2015; Böing et al., 2014; Brennan et al., 2020; Monguió‐Tortajada et al., 2019; Muller et al., 2014; Pang et al., 2020). Depending on size differences between target analytes and possible contaminations, successful SEC depends on the choice of an adequate gel matrix, path length and appropriate flow rate. For the separation of EVs and HDL particles, simple short columns with a path length of 10–30 cm that can be operated by gravity flow, may give decent results (Böing et al., 2014; Monguió‐Tortajada et al., 2019; Pang et al., 2020). But for the separation of EVs and similar‐sized ApoB‐ harbouring lipoproteins, more elaborate SEC techniques are required. S6‐FPLC has excellent separation characteristics for separation of lipoproteins (Figure 1a) (März et al., 1993; Wiesner et al., 2009). Furthermore, Superose 12 and Superose 6 have been used in EV separation from cell culture supernatants (Cardoso et al., 2021; Huang & He, 2017). Using a column length of 600 mm and a corresponding FPLC setup, we successfully introduced S6‐FPLC for the separation of liposomes in the size range of 100 nm from animal lipoproteins in a single step (Ngoune et al., 2016). Based on these observations, we selected S6‐FPLC to take the challenge of separation EVs from lipoproteins in human plasma or serum samples. This MS describes the basic method for S6‐SEC and shows the general practicability for EV separation.

Separation conditions were based on the manufacturer's recommendations (Napoleone et al., 2021), for example a flow rate of 0.2 mL/h resulting in a separation time of 4–6 h. With regard to the high viscosity and high (lipo‐)protein content of human serum, serum samples were diluted with RB (1:4 v/v) to achieve a total protein load <25 mg as recommended for best results. NaN_3_ was added to avoid microbiological contaminations. EDTA is used in lipoprotein preparations to complex trace metal ions that may catalyse lipid oxidation (e.g., Cu^2+^), and may be beneficial to prevent EV lipid oxidation as well. With the analytical column used (10 mm diameter) and a flow rate of 0.2 mL/min we achieved reliable and reproducible separation of EVs from the vast majority of proteins and lipoproteins. The elution profile of EVs was calibrated with isolated EVs from cell culture (Figure 1c). When intrinsic labelled CD9‐GFP EVs, isolated from cell culture by cUC combined with commercial SEC, were used, positive NTA signal was only seen in F15‐17 in fluorescent mode, whilst in scatter mode a low background signal for particles was seen in all fractions. High abundant plasma proteins and lipoproteins appeared in Fractions F22 onwards.

After calibration, the isolation of EVs from cell culture supernatant, either concentrated by cUC or UF, was investigated. Suitability of S6‐FPLC for the isolation of EVs was confirmed by NTA, TEM and Western blotting, showing the fraction F17 being mostly enriched in typical EV proteins including Alix, CD63, HSP 70 and TSG101 (Figure 2). For further characterisation of isolated particles, in situ labelling of EVs was performed using cholesterol as an essential component of EV membranes (Pfrieger & Vitale, 2018). To avoid excess of dye aggregates commonly contaminating EV samples after labelling (Simonsen, 2019), we labelled cells by adding liposomal BDPC. Since EVs are derived from intracellular membranes, liposomes which are generally taken up by endocytosis were preferred to deliver the BDPC to intracellular membranes. Cell culture supernatant was harvested and concentrated by UF to minimise impact on EV properties. In concert with the data from TEM and Western blotting, a significant fluorescence signal was observed in F16‐F18 with the maximum in F17, again supporting the concentration of EV s in these fractions (Figure 2e). Additionally, a detectable signal above the background fluorescence remained throughout the fractions F19‐F35, possibly referring to smaller particles, such as BDPC micelles and BDPC bound to hydrophobic proteins (Baumstark et al., 1990). When the fluorescence labelled vesicles from F16‐F17 were subjected to density gradient UC with calibrated densities (Baumstark et al., 1990), fluorescence was found only in the density range of 1.15–1.19 g/mL; as expected for EVs (Théry et al., 2018). Remaining fluorescent labelled liposomes would be expected in the density range <1.1, where no fluorescence above background level was detected.

Taken together, EVs from cell culture were concentrated mostly in fraction F16‐F17 with minor amounts in the fractions F15 and F18. As expected, SEC did not interfere with EVs structure and properties like size and density.

### 4.2 Potential of S6‐FPLC for the isolation of EVs from human serum and the impact of postprandial phase

The FPLC setup is usually equipped with an UV flow spectrophotometer, which was set to 280 nm to continuously monitor the course of a chromatography. Changes in OD may either be caused by absorption of aromatic amino acids in proteins or light scattering of particles. When diluted human serum of a fasting healthy volunteer was subjected to S6‐FPLC, high OD signals were recorded for the LDL and HDL size range and for proteins like albumin (Figure 3a). In contrast, reflecting the very low amount of EVs compared to natural human lipoproteins and serum proteins (Table 1), no signal could be recorded in the fractions F15‐F18. On one hand, absence of visible OD signal did indicate absence of significant amounts of lipoproteins, but did not allow for the detection of putative EVs on the other hand. When 10 mL human serum from a healthy donor were concentrated by cUC, a very faint 280 nm‐signal in F16 and F17 could be detected by OD measurement (Figure 3a). Unfortunately, the respective signals in the lipoprotein fractions increased as well, indicating enrichment of both, EV‐ and non‐EV particles by cUC. When analysing the fractions by NTA, we obtained in the fractions F16‐F17 less than 1% of a total particle number measured over all fractions, as expected (Figure 3b). Although pure EVs were concentrated in the fractions F15‐F17, as indicated by spiked fluorescent EVs, large VLDL mostly close to EVs by size, eluted from the fraction F18 onwards, with F18 containing EVs and lipoproteins.

For this study, human serum samples were used. Plasma may coagulate during the chromatography, clotting the column and destroying the SEC gel. To reduce this risk, anticoagulant has to be added to the chromatography buffer, which is used in large amounts. Furthermore, plasma contains high amounts of high molecular weight fibrinogen, which may contaminate early fractions where Evs are found, On the other hand, serum has the disadvantage, that during clot formation, EVs may be trapped, and clotting may induce release of platelet derived EVs, altering EV profile and count.

Important to mention, that in concert with previous publications and community guidelines (Théry et al., 2018), our data supported that UC used prior to SEC as described earlier (Baranyai et al., 2015), can be considered as an EV enrichment method allowing an efficient reduction of a sample volume. However, obtained pellets contain significant amounts of other proteins and putative particles/protein aggregates (Figures 2a and 3a), indicating that cUC is not suitable for EV purification. Furthermore, a certain loss in total particle number was observed for cUC as well.

S6‐FPLC allowed excellent separation of EVs from smaller lipoproteins (VLDL, LDL and HDL), that are omnipresent in fasting human serum. Serum samples taken under non‐fasting conditions may additionally contain CM, which are larger or at least similar in size as EVs. These postprandial lipoproteins accumulate in the EV specific fractions F16‐F17 (Figure 3d). Thus, whenever possible, fasting blood sampling is highly recommended to minimise contamination of EVs, however a short postprandial state 1–2 h may be a solution if fasting is not possible. Furthermore, it should be considered that in certain disease states, like widespread insulin resistance, remnant lipoproteins accumulate also in the fasting state (Irawati et al., 2016). Fasting TGs levels may be used as a surrogate parameter for large TG rich lipoproteins, and samples with increased TG should be interpreted with caution when analysing EVs. Pregnancy is a natural metabolic state that is characterised by insulin resistance and subsequently increased TG and remnant lipoproteins (Okazaki et al., 2004), which should be considered by investigating the role of EVs in the context of pregnancy or pregnancy disorders (Gong et al., 2021; Tersigni et al., 2021). EV lipids, for example ceramides, have been discussed as bioactive components of EVs (Elsherbini & Bieberich, 2018), but for lipid analysis, EVs should be essentially lipoprotein free.

A number of proteomics studies reported lipoproteins associated with EVs as summarised in EV public databases Exocarta (Keerthikumar et al., 2016), EVpedia (Kim et al., 2015), Vesiclepedia (Pathan et al., 2019). Although majority of these findings can be assigned to cross‐contaminations, as ApoB100 and ApoB48 are essential a non‐exchangeable structure core protein of VLDL/LDL and CM respectively (Hevonoja et al., 2000), a portion of lipoproteins, for example ApoE, can associate with EVs forming a corona as recently reported (Tóth et al., 2021). Although common one‐step enrichment techniques result in EVs/lipoprotein co‐isolation, where EVs are outnumbered by lipoproteins, this is important to consider by the estimation of a total particle and EV number in patients samples under health and disease conditions. Techniques allowing highly sophisticated separation of EVs and lipoproteins, such as S6‐FPLC presented here, might be helpful for correct estimation of the total number of EVs and EV associated biomarkers in a sample.

Our observations suggested that the efficiency of EV separation by S6‐FPLC from proteins and lipoproteins overcomes other matrixes tested so far, including Sepharose CL‐6B, CL‐4B and CL‐2B (Baranyai et al., 2015; Brennan et al., 2020; Ter‐Ovanesyan et al., 2021). Importantly, decisive impact on the quality of the separation have not only the material but also other parameters, such as path length and flow rate, which may be used for the improvement of SEC performance in EV separation.

### 4.3 Recovery

EVs free of particle contamination are mandatory to estimate the correct EV amount in human serum. Unfortunately, every purification step leads to a certain loss of EVs. For calculating recovery in serum samples, 1.0*10^10^ particles/mL were used to reflect the low EV number in human serum (Table 1) observed so far. Although recovery for high abundant proteins was >50% (Figure S9), recovery for EVs was <10% when 1.0*10^10^ particles were used. Recovery was similar for same amount of liposomes, but higher, when higher EV numbers were used for spiking. Thus recovery depends on the particle concentration. Similar dependency was observed when commercial SEC (IZON) was investigated. With lower path length and fraction size, concentration of particles in particle containing fractions was much higher, leading to higher recovery rates. Since recovery is much lower than estimated for plasma proteins and lipoproteins and dependent on particle load, loss of EVs is likely due to unspecific interaction with materials like sample cups, pipette tips, tubing etc. NTA in contrast seems to have a high accuracy, and NTA underestimation is not the cause of putative particle loss. For liposomes, estimating the theoretical particle number from measured liposomal PC concentration (Pütz et al., 2005) about ∼2.1E14 particles/mL were calculated, whilst 2.0 × 10^14^ particles/mL were measured by NTA, indication high accuracy of NTA particle concentration estimation.

Correct estimation of EV concentrations in human serum remains an obstacle. Fluorescent EVs derived from a CD9‐GFP transfected cell line were exclusively found in F15‐F17. Thus these particles might be used as internal standard for EV count estimation in human serum. Assuming that recovery for spiked fluorescent EVs is similar as recovery for intrinsic non fluorescent serum EVs, the number of measured spiked EVs can be subtracted from measured total EV count and with the estimated recovery for the respective sample the initial EV concentration in the serum sample can be calculated. Using this method for cancer patient serum, about 7.6 (± 4.7) × 10^10^ particles/mL were calculated. Using the same recovery and the measured EV number for anti CD9 antibody labelled EVs (Figure 5b), about 1.7 (± 0.9) × 10^10^ particles/ml were calculated for respective human cancer patient serum, which is slightly higher than estimated EV concentrations so far (Table 1/Lit). Use of fluorescent internal standards for particle concentration measurement is a promising approach and should be explored in larger cohorts in future work. S6‐FPLC as a single step purification methods should be of great aid to analyse vesicle numbers, unfortunately a significant loss of particles is observed during FPLC. Although high purity of EV samples was obtained, improving recovery may be the next challenge in further optimising S6‐FPLC.

### 4.4 Fluorescence‐linked S6‐FPLC

To increase sensitivity, we used a one‐step labelling of EVs and lipoproteins in an unprocessed serum sample, which we referred as a fluorescence‐linked S6‐FPLC (FL‐S6‐FPLC). Specifically, ApoB was used as a lipoprotein marker and CD9 and CD63 as EV marker (Figure 5a). We demonstrated that whilst the labelled EVs are located in the fractions F16‐F18, the unbound antibodies are separated in F28‐F35 in (Figure S7), and fluorescent antibody labelled particles were only detected in F15‐F17 in NTA analysis (Figure 5a, enlargement window 5b). Consistent results were obtained by using EVs from the cell culture supernatants and serum, supporting the feasibility and reliability of such as simple and efficient method for the localisation of EVs in the SEC fractions. It allows labelling and analysis of different species in a single step that only needs the addition of respective fluorescently labelled antibodies with different fluorophores prior to S6‐FPLC. When human serum of a healthy volunteer was used, no fluorescence was detected in F17, neither anti‐CD63‐signal nor anti‐ApoB‐fluorescence, even when 10 mL serum were concentrated by cUC. In contrast, high signals of ApoB fluorescence were recorded from F18 onwards. These data are consistent with label‐free separation and support the very low amounts of EVs in blood of healthy subjects compared to lipoproteins and further confirmed the earlier notion, that F16‐17 contain no or minute amounts of lipoprotein contamination. In contrast, significant CD63 and CD9 fluorescence was detected in serum samples from pancreatic cancer patients that had confirmed high serum levels of CD63 and CD9 in F16‐F18 (Figure 5a, enlargement window 5b), indicating the successful labelling of respective CD63‐ and CD9‐ positive EVs fluorescent‐linked S6‐FPLC in human serum samples. NTA in fluorescence mode allowed detection of fluorescence labelled particles in the fractions F15‐F17. Particle numbers were similar in fluorescence and scatter mode indicating that majority of detected EVs in these samples contained CD9.

Thus, FL‐S6‐FPLC may be a fast and convenient method for the detection of EVs harbouring a biomarker of interest among the SEC fractions. The usefulness of this method should be evaluated further in a larger cohort.

### 4.5 Limitations

Quantitative data may vary with different matrices or patient samples, for clinical data the use of internal standards would be recommended. Cell culture derived CD9‐GFP positive EVs in combination with NTA in fluorescence mode may be an excellent choice. This manuscript demonstrates the usefulness of the S6‐FPLC and FL‐S6‐FPLC for EV analysis in blood samples, however, the number of patients and healthy donors used is to low, to meet quantitative conclusions. The sample numbers are far too low to make any statistically significant statement. Thus the biomarker analysis so far should be regarded as informative but not reliable by means of differences between patients and healthy test persons.

## 5 CONCLUSIONS

- FPLC using Superose 6® can separate EVs from the majority of bulk proteins and lipoproteins when appropriate path length is given.
- Blood sampling in fasting state is highly preferable to avoid EV contamination with large lipoproteins
- Assessment of protein, EV, and lipoprotein content is mandatory for identification of EV‐enriched/protein‐ and lipoprotein‐poor fractions by the separation of blood samples using SEC. For particle counting, absence of lipoproteins is strongly recommended.
- Fluorescence‐linked FPLC is a useful method for a fast detection of EV‐containing fractions in a single step analysis
- Spiking of serum samples with fluorescence labelled EVs as internal standard might be a promising method to estimate particle counts in (human) blood samples, however dependence of the recovery rate from the concentration of particles should be considered.

## AUTHORS CONTRIBUTION

**Jerome Nouvel**: Formal analysis (equal); investigation (equal). **Gonzalo Bustos‐Quevedo**: Formal analysis (equal); investigation (equal). **Tony Prinz**: Investigation (equal); methodology (equal). **Ramsha Masood**: Formal analysis (equal); methodology (equal). **George Daaboul**: Formal analysis (equal); methodology (equal). **Tanja Gainey‐Schleicher**: Formal analysis (equal); methodology (equal). **Uwe Wittel**: Formal analysis (equal); methodology (equal). **Sophia Chikhladze**: Data curation (equal). **Bence Melykuti**: Formal analysis (equal); methodology (equal). **Martin Helmstaedter**: Methodology (equal). **Karl Winkler**: Project administration (equal). **Irina Nazarenko**: Project planing (equal); Project lead (equal); Manuscript writing (equal). **Gerhard Pütz**: Project planing (equal); Manuscript writing (equal).

## Supporting information



Supporting Information
