# Supplementary material for: Separation of small extracellular vesicles (sEV) from human blood by Superose 6 size exclusion chromatography
Source: J Extracell Vesicles. 2024 Oct 23;13(10):e70008. doi: 10.1002/jev2.70008 (PMC11497763; doi:10.1002/jev2.70008)
Supplement: Supplementary file 1 — Supporting Information [file JEV2-13-e70008-s001.pdf]

Supplementary Figure S1
Flow chart for EV isolation and analysis

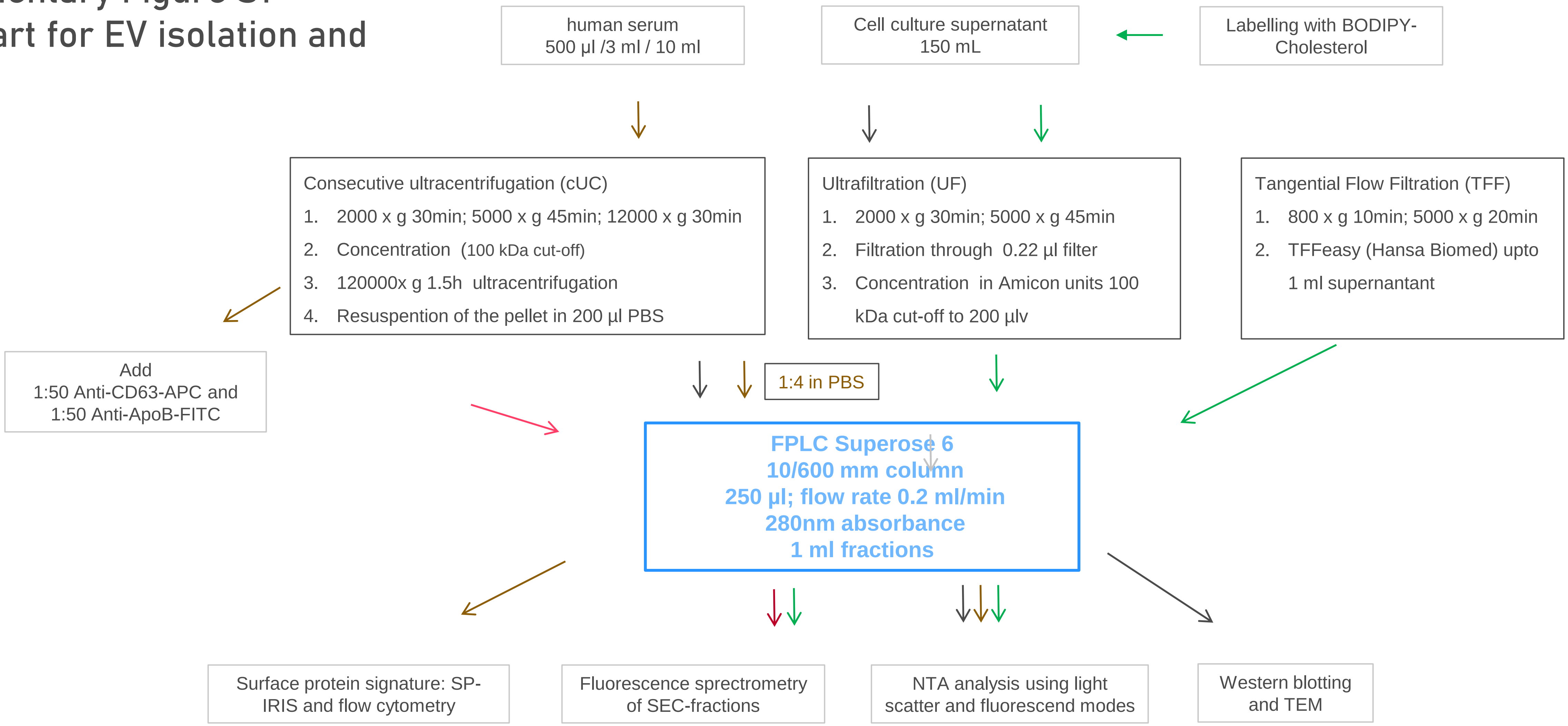

Arrows show the workflow of EV isolationand respective analysis from human serum samples (brown), cell culture supernatant from unlabelled (black) and labelled cells (green) is given with most important details. Respective serum samples were labelled by adding fluorescent antibodies (red arrows) for fluorescence-linked FPLC.

Supplementary Figure S2
Quality control of HT1080-CD9-GFP EVs after isolation by size exclusion chromatography (SEC)

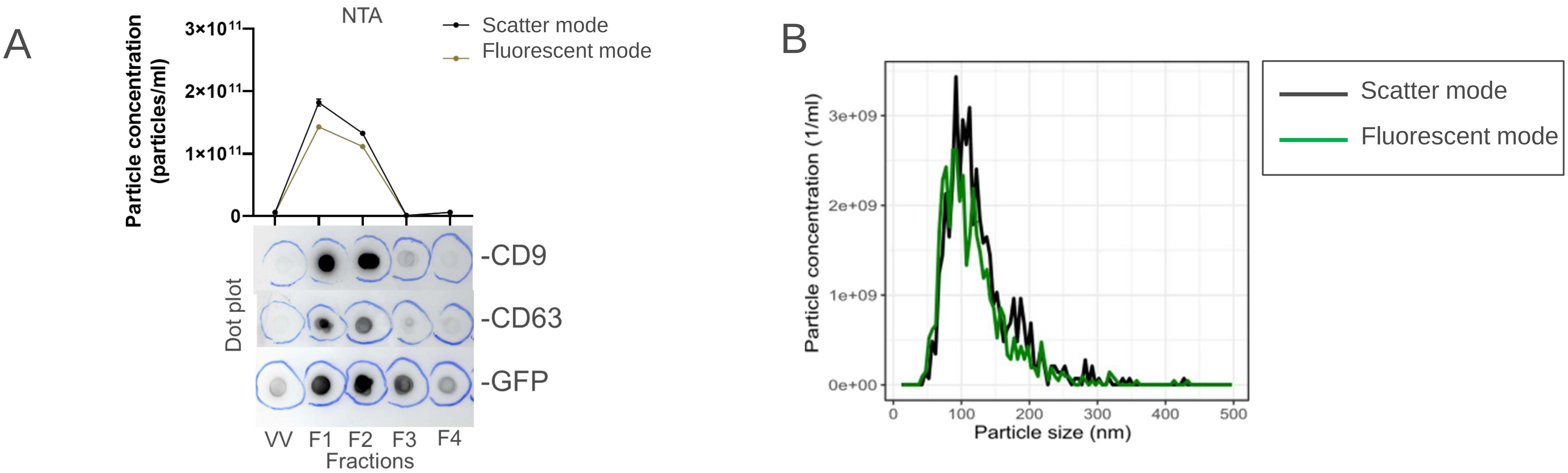

Characterization of reference EVs derived from HT1080-CD9-GFP. A) NTA and dot plot analysis of fractions aquired after using size exclusion chromatography (SEC) performed using qEV 35nm Generation 2 (IZON). Fractions F1-F2 containing maximal particle number and EV proteins CD9, CD63, and GFP fuse with CD9 were collected, pooled and concentrated using X-spinners (100 kDa cut-off) **B)** Particle size distribution by NTA in scatter and fluorescence mode of EV preparation after concentration of EV-enriched fractions.

To control the qualify of EVs derived from the HT1080-CD9-GFP cell line, dot blot was performed to control the presence of both, CD9 and GFP on EVs. Briefly, 3-5µL of each fraction were dropped on a nitrocellulose membrane 0.22µm (Cytiva) allowing them to dry. Once the sample was dried, the membrane was blocked with 5% skim milk (Powdered milk, ROTH) in TBS supplemented with 0.1% Tween (Tris Buffer Solution Tween, TBST) for 1 h. Then, the membrane was incubated overnight with the primary antibodies: CD63, CD9 or GFP, followed by three washing steps with TBST and incubation with a secondary antibody for 1 h. To visualize the dots, ECL solution (Pierce ECL western blotting substrate, Thermo Fisher) was added on top of the membrane, allowing the reaction for a few seconds, and then developing the membrane. The chemiluminescence signal was acquired by the imager (Fusion FX, Vilber).

Antibodies used were GFP 1:2000 (pabg1, Chromotek), CD9 1:5000 (D8O1A, Cell Signaling Technologies), CD63 1:2000 (Cell Signalling Technologies), anti-rabbit IgG conjugated with HRP 1:10000 (Jackson Immuno Research). All the antibodies were prepared in 5% skim milk in TBST.

Supplementary Figure S3
Recovery of high abundant serum proteins in S6-FPLC

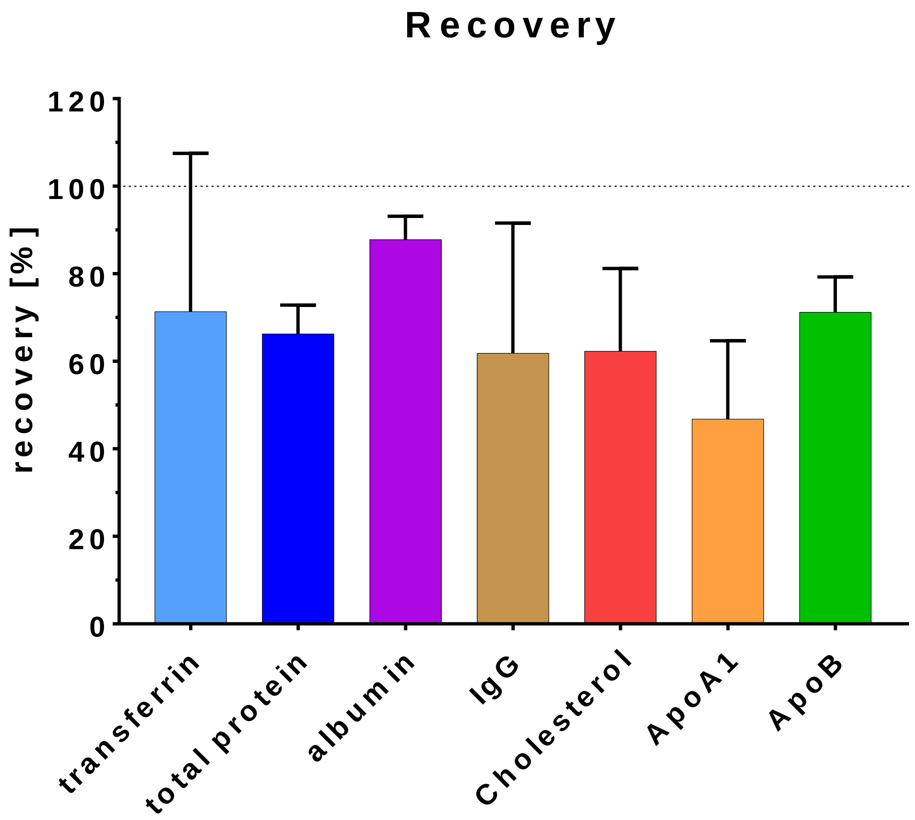

To calculate recovery, diluted serum samples were measured and observed concentrations above LOQ were summarized. Two S6-FPLC runs were combined and fractions were concentrated on Amicon® Ultra – 2 ml Ultracell-10k centrifugal filter (Merck-Millipore).
Cholesterol, lipoproteins, and typical serum proteins were measured on standard clinical chemical essay (Roche Cobas ®). Values below LOQ were set to zero. Since values below LOQ were not regarded, recovery may be slightly underestimated (n=3).

UNIVERSITÄTS KLINIKUM FREIBURG

Supplementary Figure S4
Calibration of the NTA instrument with silica nanoparticles

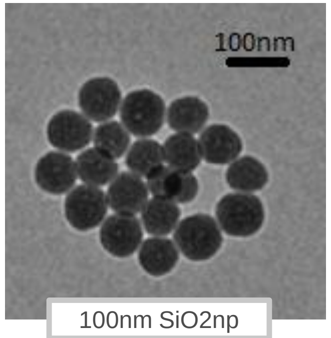

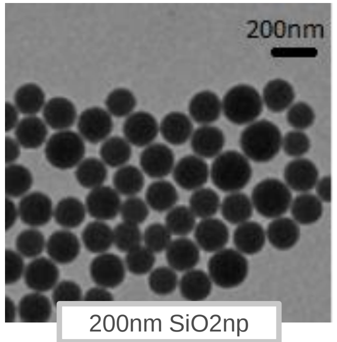

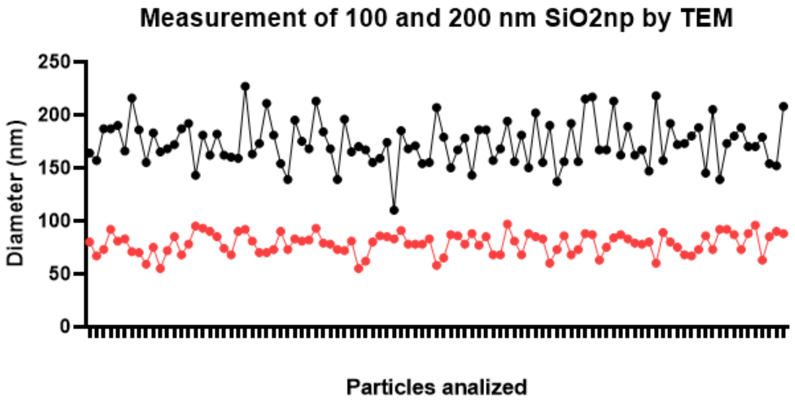

Application of SOP_EVs for the measurement of 70, 100, and 200nm SiO2np

SOP used	SiO2 np	Diameter mean (nm)	SD	Error precision factor
SOP_EVs	70 nm	74,63	3,39	3,42
	100 nm	97,82	1,91	1,11
	200 nm	186,6	5,53	0,36

$$\text{Error precision factor} = \frac{\textit{Stock concentration (given by the provider}}{\textit{Concentration measured}}$$

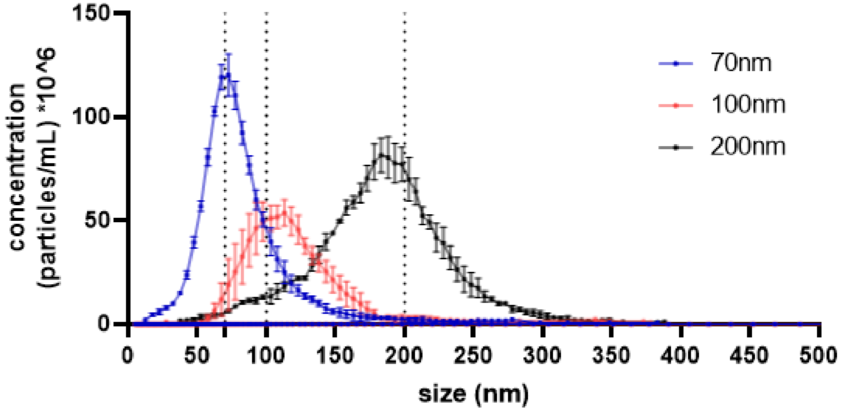

Application of SOP100_EVs for the measurement of a 1:1 ratio mixture of 100 and 200nm SiO2 nanoparticles

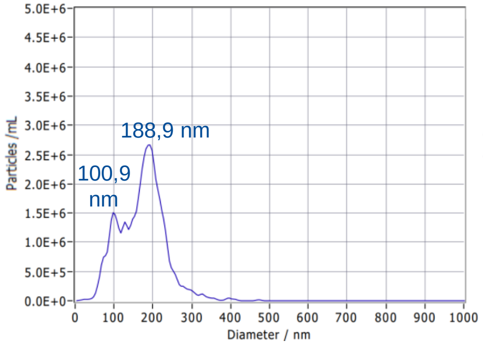

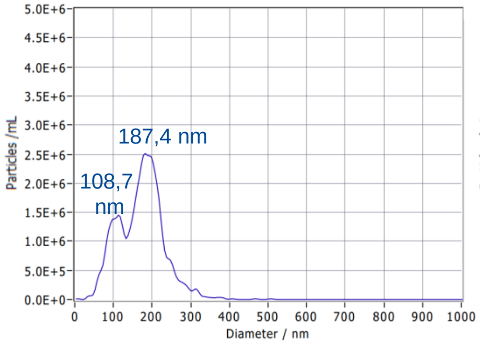

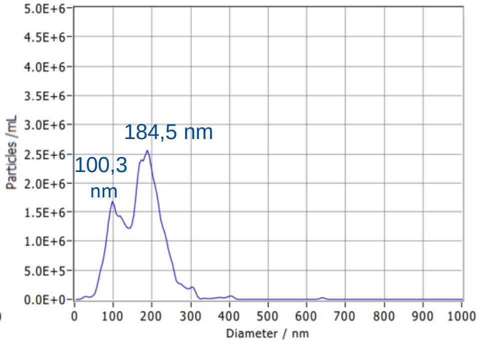

- Measurements were made using SOP100_EVs
- The sample analized was a mixture of SiO2 np of 100 nm and 200 nm in a 1:1 ratio
- The ratio of the mixture was calculated considering the absolute number of particles/ml (same absolute number of particles/ml for particles evaluated in a ratio 1:1)

UNIVERSITÄTS KLINIKUM FREIBURG

Supplementary Figure S5
Representative TEM images of S6-FPLC fractions containing no putative EVs.

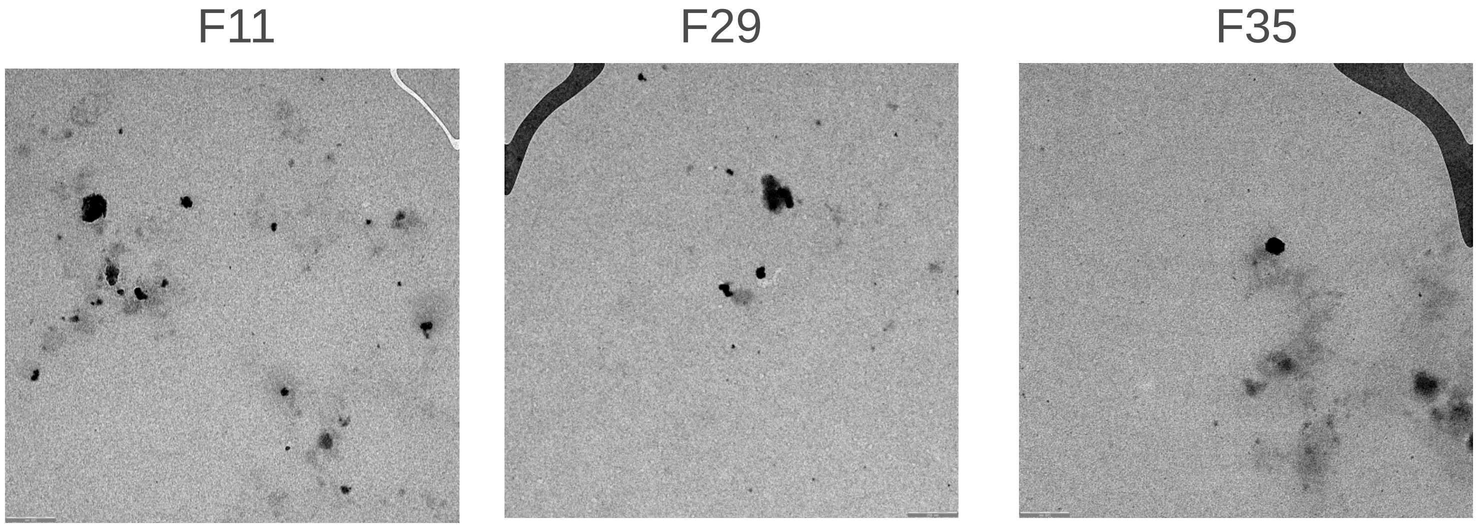

Cell culture supernatant was enriched by cUC and pellets were subjected to S6-FPLC. Pictures show typical examples of TEM images of diffrent fractions F11, F29, F35 where no EVs where expected.

Supplementary Figure S6
Detection of Calnexin in EV fractions isolated from the conditioned cell culture supernantant using Western Blotting

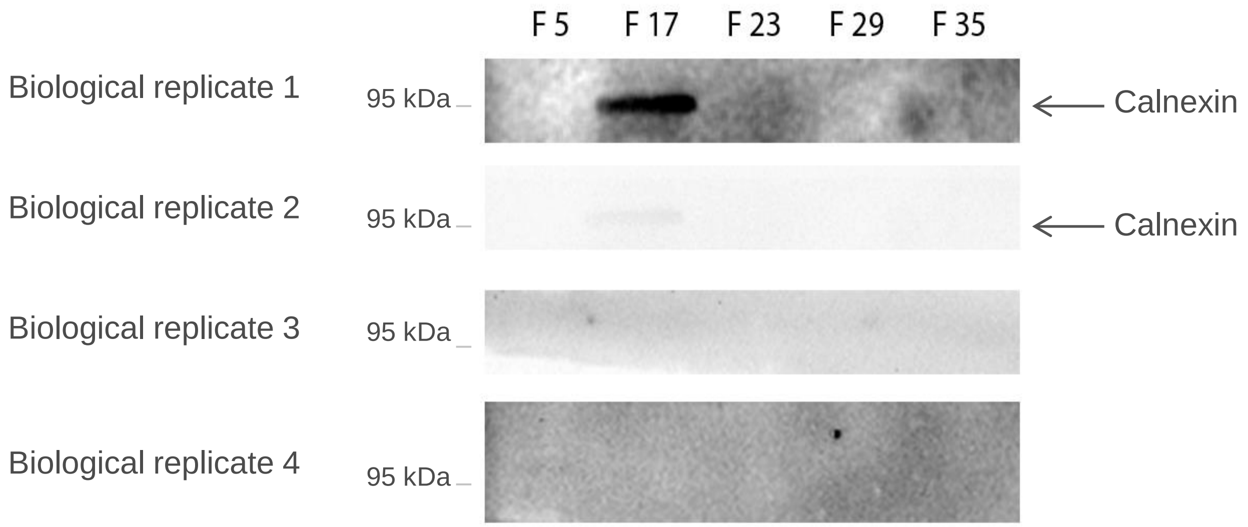

Thus EVs and apoptotic bodies are not distinguished by S6-FPLC.
When analyzing EVs from the cell cultures containing significant portions of apoptotic cells, Calnexin was detected in the fraction 17 together with the EVs markers (Suppl. Fig. S3).

Cell culture supernatant from different preparations of MDA-MB-361 cells was concentrated by UC and subjected to S6-FPLC. Representative samples were investigated by western blotting for several biomarker, including Calnexin, which is shown by the representative band at ~95 kDa (see methods for details). Depending on the quality of cells, calnexin was present when apoptotic cells were present, but absent when vital cells were used as source for EVs.

Supplementary Figure S7
Particle count and anti-CD-63-APC fluorescence in MDA-MB 361 cell culture supernatant

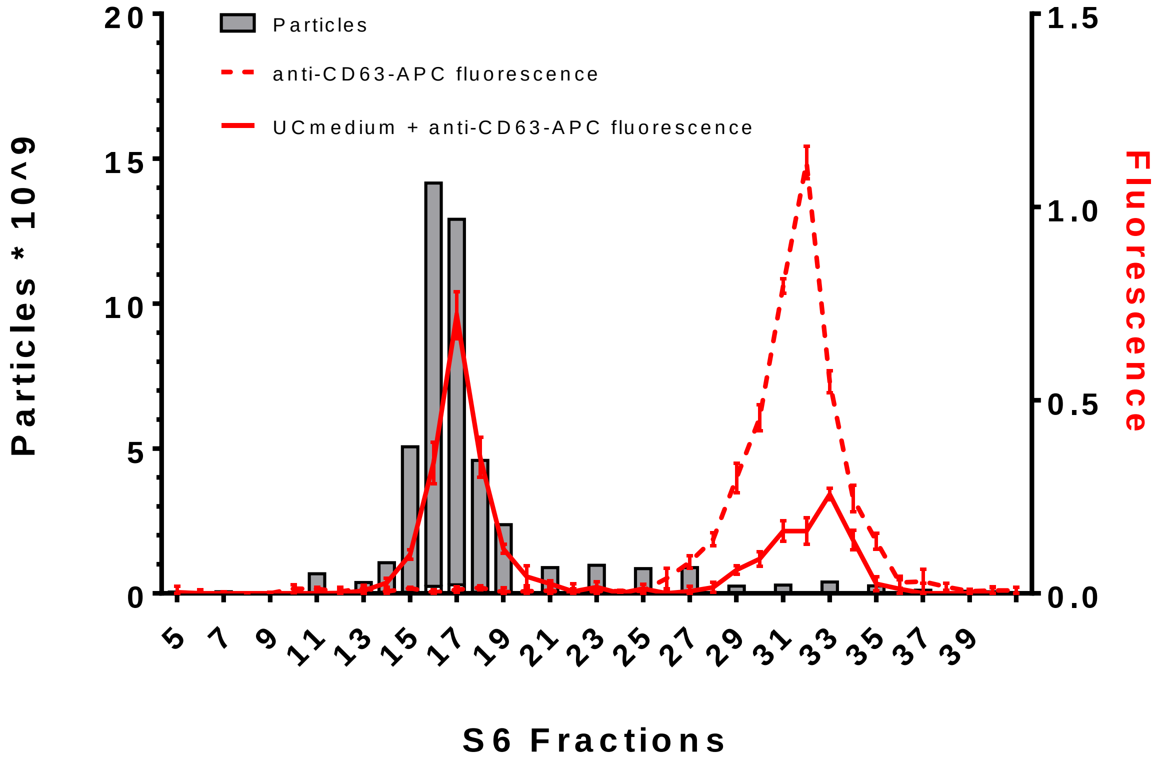

Cell culture supernatant was concentrated by UC and incubated with anti-CD-63-APC fluorescent antibody prior to S6-FPLC (see methods). Fluorescence (solid red line) and particle count measured by NTA (grey bars) are given as a representative example (n=4). Antibody without medium was used as control (dotted red line). Unbound antibody is found in F29-35, while particles and particle bound anti-CD-63 are collocated in F15-F19, peaking in F16/17.

Supplementary Figure S8
acute phase soluble proteins in cancer patients measured by MSD

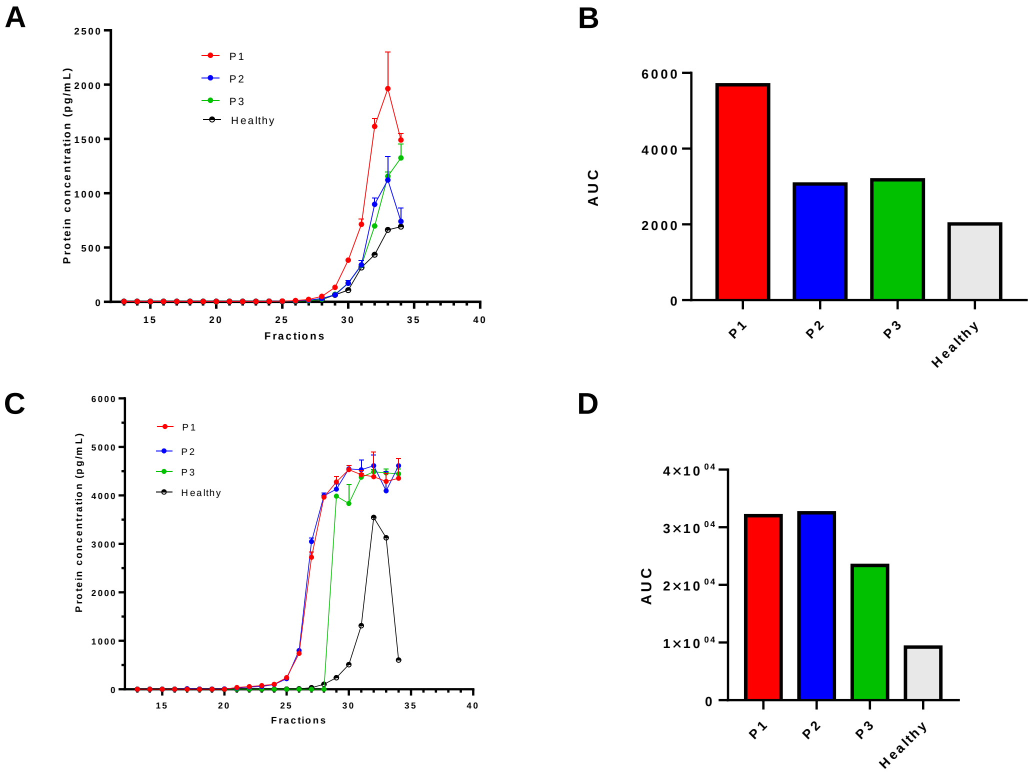

Human serum was subjected to S6-FPLC and the resulting fractions were analysed for Factor VII (A/B) and CRP (C/D) by MSD. A/C: distribution after S6-FPLC; B/D: AUC of A/C. As expected Factor VII and CRP distribute as plasma proteins in F25 onward. AUC in cancer patients is significantly higher than AUC of a healthy control. For that purposes Meso Scale Discovery (U-PLEX, MSD assay) was performed on the fractions obtained by FPLC and the unprocessed plasma sample. Two pancreatic cancer patients were used and one healthy donor as a control. Briefly, the protocol was followed as it is described by the supplier, using following modification: the fractions obtained from FPLC were used without dilution, while the plasma samples were diluted 1:4000.

Supplementary Figure S9
Particle loss during cUC enrichment of sEVs from human serum samples

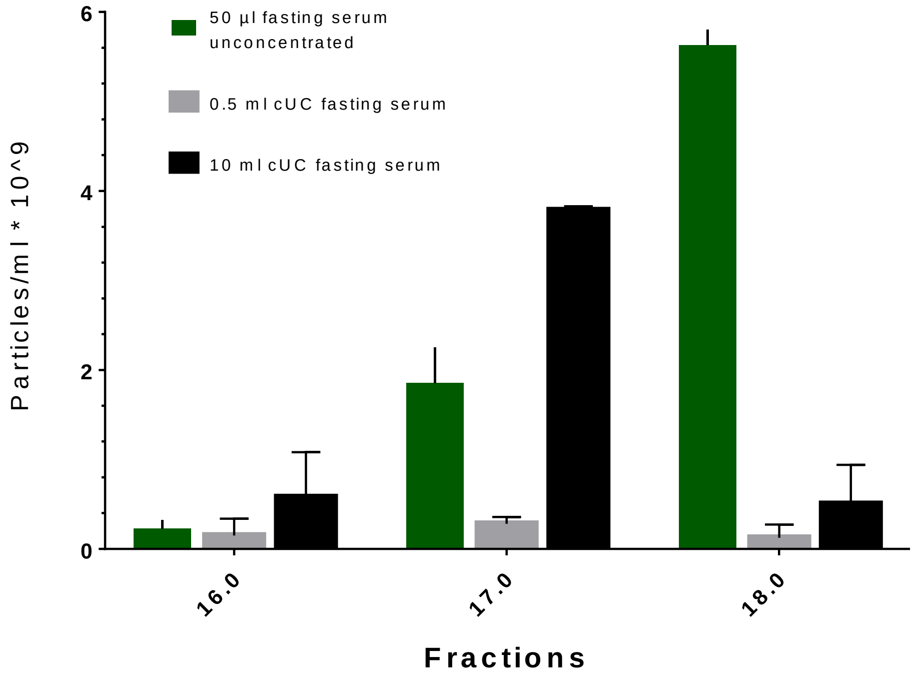

Human serum was subjected to S6-FPLC and the particle count in EV containing fractions was measured by NTA (scatter mode). Green bar: 50 µl human serum diluted in 200 µl PBS; grey bar: 0.5 ml human serum were concentrated by cUC and respective pellet was resuspended in 250 µl PBS; black bar: 10 ml human serum were concentrated by cUC and respective pellet was resuspended in 250 µl PBS; n=3

Supplementary Table ST1: Lipid particles in human plasma

Characteristics and estimated concentrations of human lipoproteins and extracellular vesicles (EVs), adopted from [9]. HDL – high density lipoprotein, LDL – low density lipoprotein, VLDL – very low density lipoprotein, CM chylomicrons and chylomicron remnants [19].

Species	Size (nm)	Density [g/ml]	characteristic apolipoprotein [kDa]$	Associated apolipoproteins	Estimated particle conc. [particles/ml]
**HDL**	5-15	1.063-1.210	ApoA-1 (28.1)	C	$8*10^{15}$
**LDL**	18-25	1.006-1.063	ApoB-100 (512)	E	$1*10^{15}$
**VLDL**	30-80	0.94-1.006	ApoB-100 (512)	C, E	$1*10^{13}$
**CM (fasting)**	>75	<0.93	ApoB-48 (242)	A,C,E	$1*10^{13}$
**CM (post-prandial)**	75-1200	<0.93	ApoB-48 (242)	A,C,E	$2*10^{13}$
**EV´s**	30-1000	1.08-1.21	N/A	(E?)	~ 10^{7}-10^{9}

$Estimated molecular weights do not include carbohydrate contributions and other post-translational modifications.

Supplementary Table ST2: Antibodies used in the study

Antibody	Dilution	Company	Catalog number
HSP70/HSC70	1/500	Santa Cruz	sc-24
tsg101	1/100	Santa Cruz	sc-7964
CD63	1/100	BD Pharmingen	556019
Alix	1/100	Cell signaling	2171s
ApoB	1/1000	Abcam	ab20737
goat anti-mouse HRP	1/1000	Biolegend	405306
goat anti-rabbit HRP	1/2000	Dako	P0448
CD63-APC	1/50	Biolegend	353007
ApoB-FITC	1/50	Abcam	ab27637
Anti-hCD63 PE	1/50	Biolegend	353003
Alex Furo 594 anti hEpCam/ CD326 PE	1/20	Biolegend	324228
Anti-hTspan8 PE	1/2	R&D Systems	FAB4734P
HSP70/HSC70	1/500	Santa Cruz	sc-24
tsg101	1/100	Santa Cruz	sc-7964
CD63	1/100	BD Pharmingen	556019
Alix	1/100	Cell signaling	2171s
ApoB	1/1000	Abcam	ab20737
goat anti-mouse HRP	1/1000	Biolegend	405306
goat anti-rabbit HRP	1/2000	Dako	P0448
CD63-APC	1/50	Biolegend	353007
ApoB-FITC	1/50	Abcam	ab27637
Darpin anti-EpCam Cy 3.5	1/5		
hCD63 Elisa kit		MyBioSource	MBS289511
hCD9 Elisa kit		MyBioSource	MBS1605956
hCD41 Elisa kit		MyBioSource	MBS3803066
hEPCAM Elisa kit		MyBioSource	MBS032831
h ApoA1(HDL) Elisa kit		R&D Systems	DAPA10
h ApoB (LDL) Elisa kit		R&D Systems	DAPB00

UNIVERSITÄTS KLINIKUM FREIBURG
